# Perinatal Endocrine–Cardiac Axis: A Narrative Review of Long-Term Cardiovascular Risks in Women with Gestational Diabetes, Hypertensive Disorders, and Thyroid Dysfunction

**DOI:** 10.3390/biomedicines14061322

**Published:** 2026-06-10

**Authors:** Ying Xie, Beiyan Chen, Shuang Gao, Jianuo Li, Bin Chen, Jieru Han

**Affiliations:** 1 School of Basic Medical Sciences, Heilongjiang University of Chinese Medicine, Harbin 150040, China; xieying@hljucm.edu.cn (Y.X.); 13009855151@163.com (J.L.); cbwww0129@126.com (B.C.); 2 Graduate School, Heilongjiang University of Chinese Medicine, Harbin 150040, China; 17323862169@163.com (B.C.); 13111547745@163.com (S.G.)

**Keywords:** gestational diabetes mellitus, hypertensive disorders of pregnancy, preeclampsia, thyroid dysfunction, postpartum thyroiditis, cardiovascular disease, women’s health, placenta, endothelial dysfunction, insulin resistance

## Abstract

**Purpose**: To review the long-term cardiovascular risks associated with three common perinatal endocrine disorders—gestational diabetes mellitus (GDM), hypertensive disorders of pregnancy (HDP), and thyroid dysfunction (including postpartum thyroiditis)—and to identify opportunities for early risk stratification and prevention. **Materials and Methods**: We conducted a structured literature search of PubMed and Web of Science for peer-reviewed articles published between January 2000 and December 2025. Search terms included combinations related to GDM, HDP, thyroid dysfunction, and cardiovascular disease (CVD). We prioritized prospective cohort studies, meta-analyses, systematic reviews, and major clinical guidelines. Key findings were synthesized thematically. **Results**: GDM is associated with a 1.6- to 2-fold increased risk of future CVD, HDP with a 1.8-fold increase, and subclinical hypothyroidism with a two-fold increase. These risks persist for decades, are independent of traditional risk factors, and are amplified by obesity, recurrence, and social determinants of health. Converging pathophysiological mechanisms include persistent insulin resistance, chronic low-grade inflammation, endothelial dysfunction, autonomic dysregulation, epigenetic modifications, and subclinical myocardial remodeling. The placenta serves as a central endocrine–cardiovascular interface, releasing anti-angiogenic factors, pro-inflammatory cytokines, and exosomal microRNAs. Despite this evidence, postpartum screening uptake remains below 50%, care is fragmented, and pregnancy history is not incorporated into CVD risk calculators. **Conclusion**: A life-course approach integrating structured postpartum screening (6–12 weeks and annually), lifestyle interventions, targeted pharmacotherapy, and multidisciplinary cardio-obstetrics programs is urgently needed to reduce the global burden of premature heart disease, stroke, and heart failure in women.

## 1. Introduction

CVD is the leading cause of death in women globally, yet it remains underrecognized in young and middle-aged women, many of whom lack traditional risk factors. The perinatal period—pregnancy and the first year postpartum—has emerged as a critical window for identifying future CVD risk. Pregnancy imposes substantial physiological challenges, including increased cardiac output, insulin resistance, and altered thyroid function. In women with limited metabolic or vascular reserve, these adaptive demands can unmask latent dysfunction, leading to complications such as GDM, HDP, and thyroid dysfunction (including postpartum thyroiditis). Importantly, these conditions are not transient events but powerful predictors of long-term CVD. A history of GDM is associated with a 63% higher odds of future CVD (OR 1.63, 95% CI 1.02–2.62) [1]; HDP with an 80% higher risk (RR 1.80, 95% CI 1.67–1.94) [2]; and subclinical hypothyroidism with a more than three-fold higher risk of heart failure (HR 3.25, 95% CI 1.96–5.39) [3]. The concept of pregnancy as a “stress test” for future cardiovascular health captures this paradigm. Women who develop GDM, HDP, or thyroid dysfunction during pregnancy reveal an underlying predisposition to cardiometabolic disease that persists years after delivery, including sustained insulin resistance, endothelial dysfunction, and subclinical myocardial remodeling [4,5,6]. This review focuses on three common perinatal endocrine disorders: GDM, HDP, and thyroid dysfunction [7,8,9]. Each has been independently linked to future myocardial infarction, stroke, heart failure, and cardiovascular death.

Despite this evidence, these conditions remain systematically overlooked, creating what we term the “overlooked double burden”. The first burden is the diagnosed endocrine disorder, typically managed during pregnancy but then abandoned after delivery. The second burden is latent cardiovascular damage—increased arterial stiffness, elevated inflammatory markers, left ventricular diastolic dysfunction—that progresses silently for years. Fewer than 50% of women attend recommended postpartum screening, primary care providers often fail to ask about pregnancy complications, and current CVD risk calculators do not include pregnancy history [10,11,12]. This double burden disproportionately affects low-income and minority women, exacerbating existing disparities.

Therefore, the aim of this narrative review is three-fold: (1) to summarize the epidemiological evidence linking GDM, HDP, and thyroid dysfunction (including postpartum thyroiditis) to long-term CVD; (2) to examine the shared pathophysiological mechanisms—including persistent insulin resistance, chronic inflammation, endothelial dysfunction, epigenetic modifications, and subclinical myocardial remodeling—that underlie these associations; and (3) to highlight current gaps in postpartum screening and care, and to propose a life-course framework for early cardiovascular risk stratification and prevention. Recognizing and addressing both burdens through structured postpartum transition, routine screening, and life-long surveillance is essential to prevent premature CVD in millions of women worldwide.

This narrative review is based on a structured literature search of PubMed and Web of Science for articles published between January 2000 and December 2025. Search terms included combinations of “gestational diabetes mellitus”, “hypertensive disorders of pregnancy”, “thyroid dysfunction”, “postpartum thyroiditis”, and “cardiovascular disease”. We prioritized prospective cohort studies, meta-analyses, and major clinical guidelines. Conference abstracts, case reports, and non-English articles were excluded. Key findings were synthesized thematically by disorder. Given the narrative nature of this review, we did not perform formal quality scoring but explicitly noted methodological limitations (e.g., residual confounding, loss to follow-up) when discussing individual studies.

## 2. GDM and Long-Term Cardiovascular Risk

### 2.1. Definition, Diagnostic Criteria, and Epidemiology

GDM is defined as glucose intolerance with onset or first recognition during pregnancy [13]. While the condition typically resolves after delivery, it identifies a subset of women with underlying β-cell dysfunction and insulin resistance that predisposes them to future cardiometabolic disease. Diagnostic criteria for GDM have varied globally, with the Carpenter-Coustan (CC) criteria identifying more cases than the National Diabetes Data Group (NDDG) criteria; women diagnosed solely by CC criteria have been shown to carry higher risks of adverse pregnancy outcomes, raising ongoing questions about optimal diagnostic thresholds [14].

**Figure 1:**
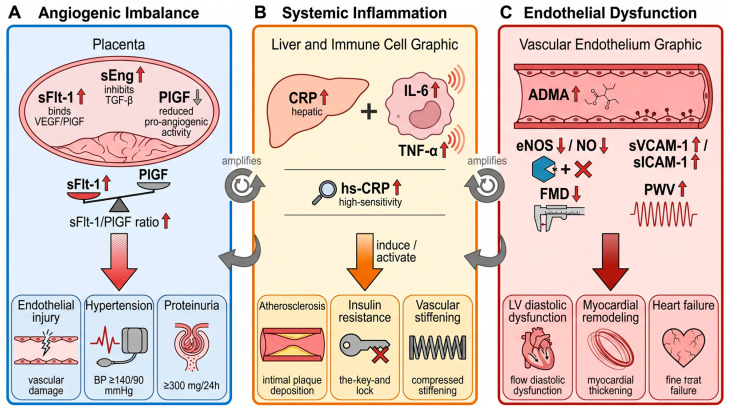
Pathophysiology of HDP: three interconnected processes. (**A**) Angiogenic imbalance—Elevated sFlt-1 and reduced PlGF (↑sFlt-1/PlGF ratio) lead to endothelial injury, hypertension, and proteinuria. (**B**) Systemic inflammation—Increased CRP, IL-6, and TNF-α promote atherosclerosis, insulin resistance, and vascular stiffening. (**C**) Endothelial dysfunction—Elevated ADMA, reduced *eNOS*/NO bioavailability, impaired FMD, increased adhesion molecules, and higher PWV drive left ventricular diastolic dysfunction, myocardial remodeling, and future heart failure.

Epidemiologically, GDM affects approximately 14% of pregnancies globally, with wide interquartile ranges (9–25%) depending on population characteristics and diagnostic standards (Figure 1). In a U.S. population-based cohort of 8,127 parous women, a history of GDM was associated with a 63% higher odds of CVD (OR 1.63, 95% CI 1.02–2.62), with CVD diagnosed on average 22.9 years after the index pregnancy [15]. Among Chinese women, GDM history conferred a 4.61-fold higher risk of abnormal glucose metabolism, a 1.57-fold higher risk of hypertension, and a 3.52-fold higher risk of metabolic syndrome postpartum [16]. In South Asian women in the U.S., prior GDM was associated with a 3.2-fold increased odds of type 2 diabetes (T2D) (95% CI 1.3–7.5) [17]. These data collectively establish GDM as a highly prevalent and potent sex-specific cardiovascular risk factor.

### 2.2. From GDM to Type 2 Diabetes: The Continuum of Metabolic Dysfunction

GDM and T2D lie on a continuous spectrum of metabolic dysfunction, with GDM frequently preceding overt diabetes by years to decades. Longitudinal evidence from the Diabetes Prevention Program Outcomes Study (DPPOS) demonstrated that women with prior GDM who received metformin had a 31% reduction in T2D incidence over 2.8 years, yet their residual diabetes risk remained substantially elevated compared to women without GDM history [18]. In a Chinese cohort, women with GDM exhibited a 4.61-fold higher risk of abnormal glucose metabolism postpartum, indicating that metabolic derangements persist well beyond pregnancy [16].

Beyond glucose homeostasis, women with prior GDM show sustained abnormalities in insulin sensitivity and lipid metabolism. A prospective cohort study found that at 3.7 years postpartum, women with previous GDM had higher homeostasis model assessment of insulin resistance (HOMA-IR) (*p* = 0.022) even after body mass index (BMI) adjustment [19]. They also displayed lower high-density lipoprotein (HDL) cholesterol (95% CI −5.17 to −1.50, *p* ≤ 0.001) and higher triglycerides, establishing a pro-atherogenic lipid profile that precedes and accompanies T2D development [15]. Thus, the transition from GDM to T2D represents not a binary event but a progressive continuum of worsening metabolic dysfunction that directly impacts cardiovascular risk.

### 2.3. Direct CV Consequences Beyond Diabetes

Importantly, the cardiovascular consequences of GDM extend beyond those mediated by incident T2D, suggesting direct vascular and cardiac effects of GDM itself. Women with a history of GDM demonstrate persistent subclinical vascular dysfunction, including increased arterial stiffness and vascular remodeling. A hospital-based cohort study reported that at 3.7 years postpartum, women with prior GDM had significantly higher pulse wave velocity (PWV) (*p* = 0.009) and elevated tissue inhibitor of metalloproteinase-1 (TIMP-1) levels, indicating ongoing vascular extracellular matrix remodeling [20].

Regarding coronary calcification, while direct coronary artery calcium (CAC) data were not extensively reported in the source review, the presence of elevated oxidized LDL and pro-inflammatory markers provides a mechanistic substrate for coronary plaque development [19,21]. More direct evidence comes from cardiac structural studies: a systematic review and meta-analysis of longitudinal cohorts found that women with a history of GDM had increased left ventricular (LV) mass and impaired LV relaxation independent of subsequent T2D, with adverse structural changes detectable as early as 20 years after the index pregnancy [5]. These findings of diastolic dysfunction and LV remodeling are particularly concerning, as they precede overt heart failure and are associated with adverse cardiovascular outcomes independent of coronary artery disease.

### 2.4. Mechanisms: Insulin Resistance, Hyperglycemia-Induced Endothelial Damage, Advanced Glycation End-Products

The pathophysiological link between GDM and long-term CVD is driven by several interconnected mechanisms. First, persistent insulin resistance is a central feature. As noted in Section 2.2, women with prior GDM maintain elevated HOMA-IR years after delivery [19], which promotes dyslipidemia, hypertension, and a pro-atherogenic state. Second, hyperglycemia-induced endothelial damage occurs both during the index pregnancy and postpartum. A systematic review of 15 studies confirmed that women with prior GDM have impaired flow-mediated dilation (FMD) and increased arterial stiffness, with elevated BMI being the most consistent aggravating factor [22]. Inflammatory pathways amplify this damage: women with GDM exhibit higher levels of tumor necrosis factor-alpha (*TNF-α*) and C-reactive protein (CRP) during pregnancy and postpartum, driving vascular inflammation [21].

Third, chronic hyperglycemia in GDM promotes the formation of advanced glycation end-products (AGEs) and oxidative stress, which reduce arterial elasticity and promote atherosclerosis [13]. The renin-angiotensin-aldosterone system (RAAS) is also dysregulated, with elevated angiotensin II levels contributing to vasoconstriction and vascular smooth muscle proliferation [13,23]. These pathways are part of shared mechanisms—including oxidative stress, inflammation, and endothelial dysfunction—that link GDM, HDP, and thyroid dysfunction to future CVD, and are discussed in detail in Section 7. Epigenetic modifications induced by maternal hyperglycemia may further program long-term cardiovascular risk, as suggested by altered fetal cardiac gene expression and persistent changes in placental DNA methylation and microRNA profiles.

### 2.5. Risk Modifiers: Obesity, Gestational Weight Gain, Lactation Duration

Several factors modify the magnitude of long-term cardiovascular risk following GDM, offering potential targets for intervention. Obesity is the most consistent and powerful risk modifier. In the systematic review by Chik et al., elevated BMI was the strongest predictor of persistent endothelial dysfunction and arterial stiffness after GDM [22]. Women with both GDM and obesity have significantly higher risks of subsequent T2D, hypertension, and CVD compared to normal-weight women with GDM alone [15,17]. Conversely, weight loss and maintenance of healthy BMI substantially attenuate risk.

Gestational weight gain (GWG) above recommended levels exacerbates postpartum metabolic dysfunction. Although the source review did not provide a dedicated GWG study in the GDM section, evidence from general pregnancy cohorts (reviewed in the context of lifestyle interventions) indicates that excessive GWG independently predicts postpartum insulin resistance and weight retention, thereby amplifying GDM-associated cardiovascular risk [24,25].

Lactation duration is a protective modifier. Women with GDM who breastfeed for 6 months or longer have a significantly lower risk of subsequent T2D (HR 0.6, 95% CI 0.4–0.9) [25]. Breastfeeding improves maternal glucose and lipid metabolism, facilitates postpartum weight loss, and may reduce long-term CVD risk, representing a low-cost, high-impact intervention in this population.

Other modifiers include GDM recurrence in subsequent pregnancies, which increases cumulative metabolic stress, and concurrent HDP, which synergistically elevate cardiovascular risk [26]. These risk modifiers underscore the importance of personalized postpartum risk stratification and tailored preventive strategies (Table 1).

## 3. HDP and Maternal Cardiovascular Sequelae

### 3.1. Spectrum: Gestational Hypertension, Preeclampsia, Eclampsia, Superimposed Preeclampsia

HDP encompass a spectrum of conditions that share elevated blood pressure as a common feature but differ in severity, timing, and associated end-organ involvement. According to current international guidelines, gestational hypertension is defined as new-onset systolic blood pressure ≥ 140 mmHg and/or diastolic blood pressure ≥ 90 mmHg after 20 weeks of gestation in a previously normotensive woman, without proteinuria [27]. Preeclampsia is diagnosed when gestational hypertension is accompanied by significant proteinuria (≥300 mg/24 h) or, in the absence of proteinuria, by other maternal end-organ dysfunction (e.g., thrombocytopenia, renal insufficiency, impaired liver function, pulmonary edema, or cerebral/visual symptoms). Eclampsia refers to the occurrence of new-onset grand mal seizures in a woman with preeclampsia, representing a life-threatening neurological complication. Superimposed preeclampsia occurs when a woman with chronic (pre-existing) hypertension develops worsening blood pressure control and new-onset proteinuria or other features of preeclampsia after 20 weeks of gestation [27,28].

The prevalence of HDP varies by population and diagnostic criteria: preeclampsia alone affects 4.6–8% of pregnancies globally, while gestational hypertension accounts for an additional 5–6% [29,30]. Severe preeclampsia (systolic BP ≥ 160 mmHg or diastolic ≥ 110 mmHg) is associated with disproportionately higher maternal and fetal risks [31]. Racial and ethnic disparities are pronounced: African American women have a 2.5-fold higher risk of coronary artery disease following HDP compared to white women [32], and South Asian populations also exhibit elevated rates of both HDP and subsequent CVD [33]. Accurate classification of the HDP subtype is essential for risk stratification, as the long-term cardiovascular prognosis differs significantly across the spectrum [34,35].

### 3.2. Short-Term CV Complications: Acute Pulmonary Edema, Peripartum Cardiomyopathy

During the peripartum period, HDP can precipitate acute, life-threatening cardiovascular complications that require immediate recognition and management. Acute pulmonary edema is a common short-term complication, occurring in 2–5% of women with severe preeclampsia [36]. It results from a combination of elevated left ventricular filling pressures, increased systemic vascular resistance, and reduced plasma oncotic pressure due to proteinuria. Clinically, patients present with acute dyspnea, hypoxemia, and bilateral crackles; chest radiography reveals interstitial or alveolar edema. Prompt treatment includes oxygen supplementation, loop diuretics, and blood pressure control with intravenous labetalol or hydralazine [36,37].

Peripartum cardiomyopathy (PPCM) is a rare but serious condition defined by the development of left ventricular systolic dysfunction (ejection fraction < 45%) in the final month of pregnancy or within five months postpartum, in the absence of another identifiable cause [38]. HDP, particularly preeclampsia, is a major risk factor for PPCM. The pathophysiological link involves anti-angiogenic factors (e.g., sFlt-1) that are elevated in preeclampsia and also impair myocardial microvascular integrity [39]. A systematic review and meta-analysis reported that women with HDP have a 178% higher risk of incident heart failure (RR = 2.78, 95% CI 2.14–3.85) compared to women without HDP, with the highest risk observed in the first year postpartum [40]. While PPCM can resolve with guideline-directed medical therapy, it carries a substantial risk of persistent cardiomyopathy and future heart failure exacerbations [38,41].

### 3.3. Long-Term Risks: Chronic Hypertension, Stroke, Heart Failure, Coronary Artery Disease

The long-term cardiovascular consequences of HDP extend far beyond the immediate postpartum period, establishing a lifetime of elevated risk. A systematic review and meta-analysis of 81 cohort studies found that women with any HDP had an 80% higher risk of future coronary artery disease (CAD) (RR 1.80, 95% CI 1.67–1.94), a 66% higher risk of CAD, and a 178% higher risk of heart failure (HF) [40]. Specifically, preeclampsia was associated with a 2.87-fold higher risk of HF (95% CI 2.14–3.85). Chronic hypertension is the most common long-term sequela: data from the Norwegian Mother and Child Cohort Study (MoBa) demonstrated that 28.6% of hypertension cases diagnosed within 10 years of delivery were attributable to prior HDP [42]. In women with recurrent preeclampsia, the odds of developing chronic hypertension increase 4.5-fold (OR 4.5, 95% CI 3.1–6.5) [40,43].

Stroke risk is also significantly elevated: the same meta-analysis reported a 103% higher risk of cerebrovascular events after HDP (RR 2.03, 95% CI 1.69–2.44) [40]. Importantly, the risk of CAD and stroke persists for decades, with evidence of subclinical atherosclerosis continuing beyond 12 months postpartum [44,45]. The combination of HDP with other pregnancy complications, such as GDM, amplifies risk synergistically: women with both HDP and GDM had a 5.7-fold higher risk of postpartum hypertension compared to those with neither condition [46]. These data underscore that HDP is not merely an acute obstetric problem but a powerful predictor of premature CVD across the female life course [47,48].

### 3.4. Pathophysiology: Angiogenic Imbalance (sFlt-1/PlGF), Systemic Inflammation, Persistent Endothelial Dysfunction

The pathophysiological mechanisms linking HDP to long-term CVD center on three interrelated processes: angiogenic imbalance, systemic inflammation, and persistent endothelial dysfunction.

Angiogenic imbalance is the most well-established driver. In normal pregnancy, the placenta secretes balanced amounts of pro-angiogenic factors such as vascular endothelial growth factor (*VEGF*) and placental growth factor (PlGF), which maintain maternal endothelial health. In preeclampsia, placental ischemia and hypoxia upregulate the anti-angiogenic factor soluble fms-like tyrosine kinase-1 (sFlt-1). sFlt-1 binds to and neutralizes circulating *VEGF* and PlGF, leading to widespread endothelial dysfunction, hypertension, and proteinuria [49,50]. Elevated sFlt-1 levels have been shown to persist in some women months to years after delivery, potentially contributing to residual vascular damage [51]. Similarly, soluble endoglin (sEng), another anti-angiogenic factor, is elevated in preeclampsia and correlates with disease severity and long-term cardiovascular risk [49,52].

Systemic inflammation and persistent endothelial dysfunction are key mechanisms linking HDP to long-term CVD. Women with HDP have elevated CRP, *IL-6*, and *TNF-α* during pregnancy, which often remain higher than in normotensive controls postpartum [53,54]. This chronic low-grade inflammation promotes atherosclerosis, insulin resistance, and vascular stiffness [55]. Even after blood pressure normalizes, prior HDP is associated with impaired flow-mediated dilation (FMD), increased arterial stiffness, and elevated adhesion molecules (sVCAM-1, sICAM-1). These functional vascular abnormalities are part of shared pathways (inflammation, oxidative stress, endothelial dysfunction) detailed in Section 7. In addition [56,57,58], HDP leads to structural cardiac remodeling: increased left ventricular end-diastolic diameter, impaired diastolic function (reduced E/A ratio, elevated E/e’ ratio), and subclinical changes that persist for years, directly contributing to future heart failure and coronary artery disease (Figure 1) [59,60].

### 3.5. Postpartum Resolution vs. Residual Vascular Damage

While blood pressure typically returns to normal within days to weeks after delivery in women with uncomplicated gestational hypertension or non-severe preeclampsia, the resolution is often incomplete at the vascular level. Postpartum resolution of overt hypertension occurs in most women by 6–12 weeks postpartum, but many continue to have higher ambulatory blood pressure readings and exaggerated blood pressure responses to exercise compared to women without HDP [61]. This “masked hypertension” contributes to ongoing cardiovascular risk even when office blood pressure appears normal [62].

Residual vascular damage manifests as persistent endothelial dysfunction, increased carotid intima-media thickness (cIMT), and arterial stiffness. A prospective cohort study of women with prior preeclampsia found that cIMT remained significantly higher than in normotensive controls up to 5 years postpartum, indicating accelerated subclinical atherosclerosis [63]. Similarly, carotid-femoral pulse wave velocity (PWV), the gold standard measure of aortic stiffness, remains elevated in women with a history of HDP, reflecting reduced vascular compliance [57,64]. These residual abnormalities are not benign; they independently predict future hypertension, stroke, and coronary events in multiple longitudinal cohorts [65,66].

The concept of “postpartum transition as a critical window” is particularly relevant for HDP. Current international guidelines recommend blood pressure monitoring at 6 weeks postpartum and annually thereafter, along with comprehensive screening for other cardiovascular risk factors (lipid profile, glucose tolerance, body mass index) [27,67]. However, loss to follow-up is common, with fewer than 40% of women with HDP attending recommended postpartum cardiovascular screening [68]. Multidisciplinary cardio-obstetrics clinics and digital health tools are emerging evidence-based strategies to bridge this care gap and prevent the transition from HDP to chronic CVD [69,70].

## 4. Thyroid Dysfunction During Pregnancy and Postpartum Thyroiditis

### 4.1. Physiological Changes in Thyroid Economy During Gestation

Pregnancy induces profound physiological alterations in thyroid function that are essential for normal fetal neurodevelopment and maternal metabolic adaptation. These changes are driven primarily by increased estrogen levels, human chorionic gonadotropin (hCG), and increased thyroxine-binding globulin (TBG). During early gestation, rising hCG (which has weak thyrotropic activity due to homology with TSH) transiently suppresses pituitary TSH secretion, leading to a physiological decrease in serum TSH levels, particularly in the first trimester. Simultaneously, estrogen-induced hepatic synthesis of TBG increases, resulting in elevated total thyroxine (TT4) and total triiodothyronine (TT3) levels, while free fractions (FT4, FT3) remain within the normal range but trend toward the lower end [71,72].

Renal clearance of iodine increases due to elevated glomerular filtration rate, and there is increased placental transfer of iodine to the fetus. In iodine-sufficient regions, the thyroid gland adapts by increasing iodine uptake. However, in iodine-deficient areas, pregnancy can unmask subclinical hypothyroidism. The thyroid gland may enlarge physiologically by 10–20% during gestation [73]. These physiological changes have important clinical implications: trimester-specific reference ranges for TSH and FT4 are required to accurately diagnose thyroid dysfunction in pregnancy, as applying non-pregnant norms would misclassify many healthy women [71,74].

After delivery, the thyroid gland rapidly returns to its non-pregnant state. However, the postpartum period is also a time of increased risk for thyroid autoimmunity, as the pregnancy-induced immunosuppression wanes, leading to a rebound immune activation (see Section 4.4). Understanding these normal physiological shifts is essential for distinguishing benign gestational adaptations from pathological thyroid disorders that carry long-term cardiovascular implications [75,76].

### 4.2. Overt and Subclinical Hypothyroidism: Effects on Maternal Cardiac Output, Diastolic Function, and Lipid Profile

Hypothyroidism during pregnancy, whether overt (elevated TSH with low FT4) or subclinical (elevated TSH with normal FT4), has well-documented effects on maternal cardiovascular function. Overt hypothyroidism affects approximately 0.3–0.5% of pregnancies, while subclinical hypothyroidism (SCH) is more common, affecting 2–5% depending on diagnostic criteria and population [71,77].

Thyroid hormones directly regulate cardiac contractility, heart rate, and systemic vascular resistance. In hypothyroidism, reduced triiodothyronine (T3) availability leads to decreased myocardial contractility, reduced heart rate (bradycardia), and increased systemic vascular resistance due to reduced endothelial nitric oxide synthase activity. Consequently, cardiac output falls, and diastolic function becomes impaired. Echocardiographic studies in non-pregnant hypothyroid individuals show prolonged isovolumetric relaxation time, reduced early diastolic filling (E wave), and increased reliance on atrial contraction (A wave), findings that are reversible with levothyroxine therapy [78,79]. During pregnancy, these changes may be partially masked by pregnancy-associated hemodynamic adaptations but become clinically relevant in women with untreated or undertreated hypothyroidism [80].

Hypothyroidism is associated with a classic atherogenic lipid phenotype: elevated total cholesterol, low-density lipoprotein cholesterol (LDL-C), and triglycerides, with reduced high-density lipoprotein cholesterol (HDL-C). A study of 767 adults found that SCH was associated with higher LDL-C (130 vs. 120 mg/dL, *p* < 0.05) and lower HDL-C (45 vs. 50 mg/dL, *p* < 0.05) compared to euthyroid controls [81]. These lipid disturbances persist into the postpartum period in women with undiagnosed or undertreated hypothyroidism, contributing to accelerated atherosclerosis [82]. Importantly, levothyroxine replacement therapy reduces LDL-C by 10–15% and improves other lipid parameters in both overt and subclinical hypothyroidism [83,84].

In non-pregnant populations, SCH with TSH > 7 mIU/L is associated with a 3.25-fold higher risk of heart failure events (HR 3.25, 95% CI 1.96–5.39) [85]. Among women with a history of gestational hypothyroidism, the risk of future hypertension, myocardial infarction, and stroke is similarly elevated, particularly when thyroid dysfunction persists or recurs in the postpartum period [78,80]. Longitudinal cohort data further confirm that gestational hypothyroidism is associated with a two-fold higher risk of incident CVD over 20 years of follow-up, independent of traditional risk factors [80].

### 4.3. Risk of Tachycardia, Atrial Fibrillation, and Hypertensive Disorders

Hyperthyroidism during pregnancy is less common than hypothyroidism, affecting approximately 0.2% of pregnancies. The two main etiologies are Graves’ disease (autoimmune hyperthyroidism, accounting for 85–95% of cases) and gestational transient thyrotoxicosis (GTT, also called hCG-mediated hyperthyroidism). Both conditions can have adverse cardiovascular consequences for the mother [71,86].

Excess thyroid hormones increase β-adrenergic receptor sensitivity, leading to tachycardia, increased myocardial contractility, increased stroke volume, and reduced systemic vascular resistance. The net effect is a high-output state. Clinically, women present with palpitations, heat intolerance, tremor, and anxiety. The most serious cardiac complications include supraventricular tachycardia (particularly sinus tachycardia and atrial fibrillation) and hypertensive disorders. A multi-ethnic cohort study found that hyperthyroidism was associated with an increased risk of atrial fibrillation (OR 1.8, 95% CI 1.1–2.9) [87]. Atrial fibrillation during pregnancy carries risks of thromboembolism and hemodynamic compromise, even in women without pre-existing structural heart disease [88].

Gestational transient thyrotoxicosis (GTT) occurs in approximately 1–3% of pregnancies and is caused by very high hCG levels (e.g., in multiple gestation or hyperemesis gravidarum) stimulating the TSH receptor. It typically presents with mild biochemical hyperthyroidism (suppressed TSH, normal or mildly elevated FT4) and resolves spontaneously by 14–20 weeks of gestation. Although GTT is generally benign, severe cases can lead to maternal tachycardia, weight loss, and dehydration, and there is an association with gestational hypertension and preeclampsia [89,90].

Women with Graves’ disease who require antithyroid medication during pregnancy often continue to have autoimmune thyroid dysfunction postpartum, including postpartum thyroiditis (Section 4.4). Long-term cardiovascular risks include persistent risk of atrial fibrillation, heart failure, and, if treated with radioactive iodine or surgery, subsequent hypothyroidism with its associated lipid and vascular effects [78,91]. Controlled studies indicate that even after restoration of euthyroidism, women with a history of hyperthyroidism have higher rates of hypertension and left ventricular diastolic dysfunction compared to the general population [92,93].

### 4.4. Postpartum Thyroiditis: Autoimmune Phenomenon, Biphasic Course and Potential Long-Term Thyroid–Cardiac Axis Disruption

Postpartum thyroiditis (PPT) is an autoimmune inflammatory condition of the thyroid gland that occurs within the first year after delivery, typically between 1 and 6 months postpartum. It is caused by the rebound of immune function following the physiological immunosuppression of pregnancy. Women with pre-existing thyroid autoimmunity (positive anti-thyroid peroxidase [*TPO*] antibodies) are at highest risk: approximately 50% of TPOAb-positive women develop PPT, compared to 5–10% of the general obstetric population [71,94]. PPT classically follows a biphasic pattern, though a monophasic hypothyroid or (less commonly) monophasic thyrotoxic course can occur.

Thyrotoxic phase (1–4 months postpartum): Destruction of thyroid follicles releases preformed thyroid hormones into the circulation, causing transient hyperthyroidism. Symptoms include fatigue, palpitations, heat intolerance, and anxiety. This phase typically lasts 2–8 weeks [94,95].

Hypothyroid phase (4–8 months postpartum): After the release of stored hormones is exhausted, the damaged thyroid gland produces insufficient hormones, leading to hypothyroidism. Symptoms include fatigue, weight gain, cold intolerance, constipation, and depression. This phase lasts 4–6 months in most women, but up to 20–30% of women with PPT will develop permanent hypothyroidism requiring lifelong levothyroxine therapy [71,96].

Both phases of PPT carry cardiovascular risks, though they differ in nature. During the thyrotoxic phase, women are at risk for tachycardia and, rarely, atrial fibrillation [88]. During the hypothyroid phase, the adverse lipid profile (elevated LDL-C, triglycerides) and impaired diastolic function characteristic of hypothyroidism become relevant [78,82]. Moreover, the thyroid–cardiac axis disruption may persist even after biochemical euthyroidism is restored. Women with a history of PPT have higher rates of subclinical atherosclerosis, as evidenced by increased carotid intima-media thickness and arterial stiffness, compared to women without PPT [96,97]. Additionally, thyroid autoimmunity itself (TPOAb positivity) has been independently associated with endothelial dysfunction and increased cardiovascular risk, even in euthyroid individuals.

### 4.5. Thyroid Autoimmunity as an Independent CV Risk Factor in Women

Emerging evidence suggests that thyroid autoimmunity, defined by the presence of circulating anti-thyroid peroxidase (TPOAb) and/or anti-thyroglobulin (TgAb) antibodies, may confer cardiovascular risk independently of thyroid hormone status. This is a paradigm shift from the traditional view that only overt or subclinical thyroid dysfunction affects the heart [98,99].

Thyroid autoantibodies are present in approximately 10–15% of women of reproductive age, with higher rates in those with a family history of autoimmune disease. In women who develop postpartum thyroiditis, TPOAb titers rise sharply in the first 3–6 months postpartum and may remain elevated for years [71,100].

Autoimmune-mediated chronic inflammation is the likely link. TPOAb positivity is associated with elevated levels of high-sensitivity C-reactive protein (hs-CRP), interleukin-6 (*IL-6*), and other inflammatory markers, even when TSH and FT4 are within normal limits [101,102]. This low-grade systemic inflammation promotes endothelial dysfunction, arterial stiffness, and atherosclerosis. In a study of euthyroid women, TPOAb positivity was associated with impaired flow-mediated dilation (FMD) and increased carotid intima-media thickness, independent of age, BMI, and blood pressure [103,104].

In the large population-based ELSA-Brazil study, subclinical hypothyroidism was associated with higher carotid intima-media thickness and increased risk of coronary artery disease [105]. However, even after excluding women with SCH, those with isolated TPOAb positivity had a modest but significant increase in cardiovascular events over 10-year follow-up (HR 1.3, 95% CI 1.1–1.6) [78]. Notably, one cross-sectional study paradoxically found that TPOAb positivity was associated with a lower risk of metabolic syndrome (OR 0.465, 95% CI 0.236–0.917) [106], suggesting that the relationship may be complex and influenced by age, ethnicity, and other factors. A 2019 systematic review and meta-analysis further confirmed that thyroid autoimmunity is associated with a 36% increased risk of cardiovascular events in euthyroid women [107].

Implications for women with perinatal thyroid disorders: For women who experience gestational hypothyroidism, Graves’ disease, or postpartum thyroiditis, the presence of persistent thyroid autoimmunity may indicate an ongoing heightened cardiovascular risk that extends beyond the period of thyroid dysfunction. Current guidelines recommend annual thyroid function testing for women with a history of PPT or other autoimmune thyroid diseases [71,108], but routine assessment of cardiovascular risk using traditional calculators that incorporate pregnancy history, including autoimmune thyroid disorders, is still not standard practice. Emerging research suggests that anti-inflammatory or immunomodulatory strategies may reduce TPOAb titers and potentially lower cardiovascular risk, though randomized trial evidence is still limited [109,110].

## 5. The Placenta as an Endocrine–Cardiovascular Interface

### 5.1. Placental Hormones with CV Effects: sFlt-1, Soluble Endoglin, TNF-α, Leptin, Resistin

The placenta is not merely a passive organ for nutrient and gas exchange; it functions as a major endocrine gland that actively secretes a wide array of hormones, cytokines, and angiogenic factors into the maternal circulation. These placental-derived factors have profound effects on maternal cardiovascular physiology and, when dysregulated, contribute to the pathogenesis of perinatal endocrine disorders and their long-term cardiovascular sequelae [111].

sFlt-1 is an anti-angiogenic protein that binds and neutralizes circulating *VEGF* and placental growth factor (*PlGF*). In normal pregnancy, sFlt-1 levels rise modestly toward term, maintaining a balanced angiogenic state. In preeclampsia, placental hypoxia dramatically upregulates sFlt-1 expression, leading to a marked increase in maternal sFlt-1 levels and a corresponding decrease in free *PlGF*. The resultant angiogenic imbalance causes systemic endothelial dysfunction, hypertension, and proteinuria [112,113]. Elevated sFlt-1/PlGF ratio is now used clinically as an adjunctive test for predicting and diagnosing preeclampsia, with high negative predictive value for ruling out preeclampsia within 1 week of testing [114].

Soluble endoglin (sEng) is another anti-angiogenic factor produced by the placenta that potentiates the vascular-damaging effects of sFlt-1. It inhibits transforming growth factor-β (TGF-β) signaling in the vascular endothelium, further impairing vascular integrity, reducing nitric oxide bioavailability, and promoting hypertension [52]. sEng levels rise earlier than sFlt-1 in some preeclamptic women, correlate directly with disease severity, and are independently associated with the risk of adverse maternal outcomes including pulmonary edema and HELLP syndrome [115].

In GDM, the placenta engages in bidirectional crosstalk with maternal adipose tissue, leading to increased production of pro-inflammatory cytokines and adipokines. Tumor necrosis factor-alpha (TNF-α) impairs insulin signaling via serine phosphorylation of insulin receptor substrate-1 (IRS-1) and promotes endothelial activation and vascular inflammation [116]. Leptin, which is significantly elevated in GDM pregnancies, contributes to systemic insulin resistance, sympathetic nervous system activation, and vascular smooth muscle proliferation, all of which drive long-term cardiovascular risk [117]. Resistin, also upregulated in the GDM placenta, promotes hepatic glucose production, impairs peripheral insulin sensitivity, and enhances endothelial expression of adhesion molecules that accelerate atherogenesis [118]. Together, these factors create a systemic pro-inflammatory and insulin-resistant state that persists into the postpartum period, even after glucose homeostasis normalizes [119].

**Figure 2:**
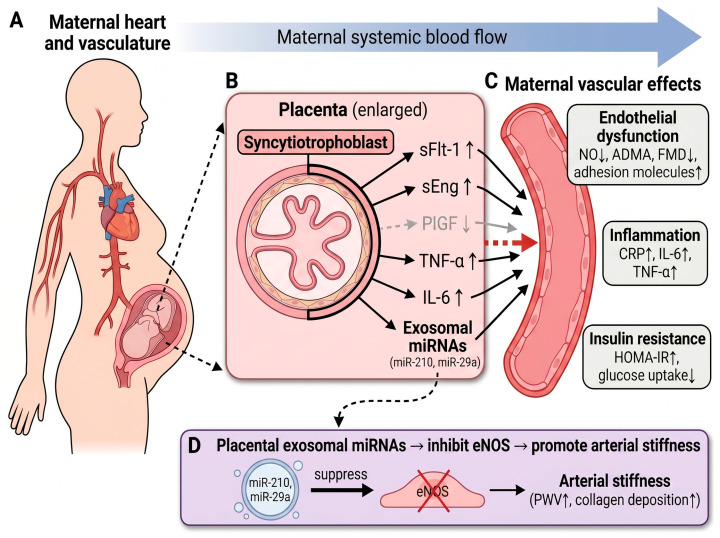
The placenta as an endocrine–cardiovascular interface: secretion of vasoactive, inflammatory, and exosomal factors that mediate maternal vascular injury. (**A**) Heart and systemic vasculature. (**B**) Syncytiotrophoblasts release anti-angiogenic proteins (sFlt-1, sEng), pro-angiogenic PlGF, pro-inflammatory cytokines (TNF-α, IL-6), and exosomes. (**C**) These factors collectively induce endothelial dysfunction, low-grade inflammation, and insulin resistance. (**D**) miR-210 and miR-29a (enriched in GDM and preeclampsia) suppress endothelial *eNOS* expression and promote arterial stiffness, contributing to long-term cardiovascular risk.

The placenta also produces human placental lactogen (hPL), a key diabetogenic hormone that antagonizes maternal insulin action and increases maternal glucose availability to the fetus; in excess, it contributes directly to the pathophysiology of GDM [120]. Additionally, placental corticotropin-releasing hormone (CRH) influences maternal hypothalamic-pituitary-adrenal (HPA) axis activity, modulates systemic vasoreactivity, and may contribute to blood pressure dysregulation in preeclampsia (Figure 2) [121].

### 5.2. Placental Thyroid Hormone Metabolism: Deiodinases and Transporters

The placenta plays a critical role in regulating thyroid hormone availability to the fetus and modulating maternal thyroid economy throughout gestation. It expresses a specific repertoire of iodothyronine deiodinases (D2, D3) and thyroid hormone transporters (e.g., MCT8, OATP1C1), which tightly control the local and systemic concentrations of biologically active T3 and inactive T4 metabolites [122].

Deiodinases: Type 2 deiodinase (D2) converts the prohormone T4 to the biologically active T3, while type 3 deiodinase (D3) inactivates T4 and T3 to reverse T3 (rT3) and T2, respectively. The human placenta expresses high levels of D3 throughout gestation, which creates a functional barrier that limits the transfer of active thyroid hormones from mother to fetus, protecting the developing fetus from excessive maternal thyroid hormone fluctuations [76,123]. Placental D2 is expressed primarily in the first trimester, ensuring local T3 production for early placental and fetal development. However, in conditions of maternal thyroid dysfunction, this protective regulatory mechanism can be overwhelmed, altering both maternal thyroid hormone status and fetal exposure [124].

Placental thyroid hormone transporters, particularly monocarboxylate transporter 8 (MCT8) and organic anion-transporting polypeptide 1C1 (OATP1C1), are essential for facilitating the passage of T4 and T3 across the syncytiotrophoblast barrier. These transporters are differentially expressed across gestation, with MCT8 expression increasing toward term to support fetal thyroid hormone accretion [125,126]. Mutations or dysregulation of these transporters can impair fetal neurodevelopment even when maternal thyroid function is biochemically normal.

In women with gestational hypothyroidism or hyperthyroidism, placental deiodinase and transporter function may be significantly altered, potentially affecting both maternal cardiovascular adaptation (via modified systemic thyroid hormone levels) and fetal development. For example, in severe preeclampsia, placental D3 expression is reduced, leading to increased transplacental T3 transfer and potential fetal thyrotoxicosis, while also modifying maternal thyroid hormone bioavailability [127]. In GDM, placental deiodinase activity is dysregulated, leading to reduced local T3 production and impaired placental insulin signaling [128]. Understanding these placental mechanisms is essential for interpreting maternal thyroid function tests during pregnancy and for predicting postpartum thyroid and cardiovascular outcomes.

### 5.3. Placental-Derived Exosomes and MicroRNAs: Intercellular Communication Affecting Maternal Endothelium

Recent advances in placental biology have identified exosomes—small extracellular vesicles (30–150 nm) released by the syncytiotrophoblast—as key mediators of intercellular communication between the placenta and the maternal vasculature. These exosomes carry a cargo of proteins, lipids, and microRNAs (miRNAs) that can be taken up by maternal endothelial cells, immune cells, and other distant tissues, thereby directly modulating maternal cardiovascular function and long-term disease risk [129].

In healthy pregnancy, placental exosomes are released into the maternal circulation in increasing quantities as gestation advances. They carry tolerogenic signals that help modulate the maternal immune system to prevent fetal rejection, promote physiological angiogenesis, and support maternal vascular adaptation to pregnancy [130].

In preeclampsia, the number, release kinetics, and molecular composition of placental exosomes are markedly altered. They contain elevated levels of sFlt-1, sEng, TNF-α, and other pro-inflammatory factors, and they directly induce endothelial dysfunction, reduce nitric oxide production, and increase vascular permeability when incubated with human endothelial cells in vitro [131,132]. Specific miRNA cargoes, such as hypoxia-induced miR-210, are highly enriched in preeclamptic exosomes and have been shown to suppress endothelial nitric oxide synthase (*eNOS*) expression, impair vasodilation, and promote vascular stiffness in preclinical models [133]. These exosomal miRNAs can be detected in the maternal circulation months before the clinical onset of preeclampsia, making them promising early predictive biomarkers [134].

In GDM, placental exosomes carry a distinct set of miRNAs that modulate maternal insulin signaling pathways. For example, miR-29a and miR-222 are consistently dysregulated in GDM exosomes, and their levels in maternal plasma correlate with the severity of insulin resistance during pregnancy [135]. These exosomal miRNAs can be transferred to maternal peripheral tissues, including adipose tissue and skeletal muscle, where they persistently alter insulin signaling and contribute to the persistence of insulin resistance postpartum [136]. Circulating exosomal miRNA signatures from GDM pregnancies have also been associated with subclinical atherosclerosis and endothelial dysfunction years after delivery, serving as potential biomarkers for future type 2 diabetes (T2D) and CVD [137].

Circulating placental exosomes and their miRNA cargo can be isolated from maternal blood as early as the first trimester, representing a promising, non-invasive source of biomarkers for predicting which women with GDM or HDP will go on to develop long-term CVD. Prospective cohort studies are ongoing to validate exosomal miRNA signatures for postpartum risk stratification, and early-phase studies are exploring exosome-targeted therapies to mitigate the maternal vascular damage associated with adverse pregnancy outcomes [138,139].

### 5.4. The “Three-Hit” Model

**Figure 3:**
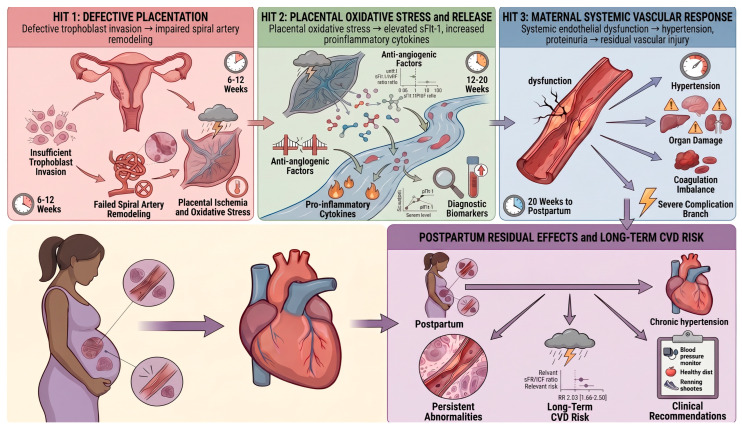
The three-hit model of preeclampsia and its link to future cardiovascular disease. Hit 1: defective trophoblast invasion → failed spiral artery remodeling → placental hypoxia. Hit 2: placental oxidative stress → release of anti-angiogenic factors (sFlt-1, sEng) and pro-inflammatory cytokines. Hit 3: systemic endothelial dysfunction → hypertension, proteinuria, coagulation abnormalities. Residual vascular damage persists postpartum, leading to elevated long-term risks of chronic hypertension, coronary artery disease, stroke, and heart failure.

The pathophysiology of preeclampsia is best understood through the “two-stage” or “three-hit” model (Figure 3, legend). This framework integrates placental, maternal, and environmental factors to explain the progression from abnormal placentation to clinical maternal disease and long-term cardiovascular sequelae [53].

Hit 1—Abnormal placentation (first trimester): Inadequate remodeling of maternal spiral arteries by invading extravillous trophoblast cells leads to reduced uteroplacental blood flow and chronic placental ischemia and hypoxia. In normal pregnancy, trophoblast invasion transforms the high-resistance, vasoactive spiral arteries into low-resistance, dilated vessels that ensure continuous placental perfusion. In preeclampsia, this transformation fails to occur, leaving the placenta vulnerable to intermittent hypoxia and reperfusion injury [140,141]. The causes of abnormal placentation are multifactorial, including immunologic maladaptation to the semi-allogeneic fetus, genetic factors, and pre-existing maternal conditions such as chronic hypertension, diabetes, or obesity.

Hit 2—Placental oxidative stress and release of anti-angiogenic factors (second trimester): The chronically ischemic and hypoxic placenta undergoes excessive oxidative stress, generating reactive oxygen species (ROS) that damage trophoblast cells, induce endoplasmic reticulum stress, and accelerate placental senescence [142]. In response, the placenta upregulates and releases large quantities of sFlt-1, sEng, pro-inflammatory cytokines (*TNF-α*, *IL-6*), and other vasoactive factors into the maternal circulation. This represents the “endocrine phase” of preeclampsia, where the placenta acts as a dysfunctional endocrine organ driving systemic maternal disease [143,144].

Hit 3—Maternal systemic vascular response (third trimester to postpartum): The circulating anti-angiogenic and pro-inflammatory factors trigger a profound systemic response in the mother: widespread endothelial dysfunction, increased vascular permeability, activation of the coagulation cascade, and heightened sensitivity to vasopressors such as angiotensin II [145]. These changes manifest clinically as hypertension, proteinuria, and, in severe cases, eclampsia, pulmonary edema, or HELLP syndrome. Importantly, even after delivery and removal of the placenta, some of these vascular changes persist, including sustained endothelial dysfunction, arterial stiffness, and epigenetic modifications of vascular cells, contributing to long-term cardiovascular risk [47,146].

Relevance to GDM and thyroid disorders: While the three-hit model was originally developed for preeclampsia, analogous pathways operate in GDM and gestational thyroid dysfunction. In GDM, maternal hyperglycemia and hyperinsulinemia induce placental oxidative stress, mitochondrial dysfunction, and inflammation (Hit 2), leading to the release of pro-atherogenic factors that drive maternal endothelial dysfunction and persistent insulin resistance (Hit 3) [147]. In gestational thyroid disorders, altered placental deiodinase activity and thyroid hormone signaling modify placental oxidative stress and the severity of maternal vascular responses to adverse pregnancy stimuli, amplifying long-term cardiovascular risk (Figure 3) [148].

### 5.5. Implications for Predicting CV Risk Based on Placental Pathology

Placental pathology obtained after delivery—whether from routine examination or clinically indicated sampling—provides a unique, underutilized window into a woman’s long-term cardiovascular health. This represents an unprecedented opportunity to integrate obstetric and cardiovascular care, identifying high-risk women at the time of delivery before the onset of clinical CVD [149]. Placental lesions associated with higher CVD risk: Several specific placental histologic findings have been consistently linked to future maternal CVD.

Characterized by decidual arteriopathy, acute atherosis, villous infarcts, and increased villous syncytial knots. Maternal vascular malperfusion (MVM) is the histologic hallmark of abnormal placentation and is highly associated with preeclampsia and intrauterine growth restriction. Women whose placentas show MVM have significantly higher rates of subsequent chronic hypertension, dyslipidemia, and CVD compared to women with preeclampsia but normal placental histology, even after adjusting for traditional cardiovascular risk factors [150,151]. A 2022 prospective cohort study found that women with severe MVM had a 3.2-fold higher risk of incident hypertension and a 2.7-fold higher risk of cardiovascular events within 10 years of delivery [152].

Including thrombi in fetal chorionic vessels, avascular villi, and villous stromal-vascular karyorrhexis. Fetal vascular malperfusion (FVM) is associated with maternal hypercoagulable states and may indicate an underlying systemic thrombophilia, which itself increases long-term CVD risk [153].

An inflammatory lesion involving maternal T-cells invading the placental villi. Chronic villitis of unknown etiology (VUE) is associated with recurrent pregnancy loss and may indicate a systemic maternal autoimmune or inflammatory diathesis that predisposes to accelerated atherosclerosis and CVD [154].

Low placental weight (<10th percentile for gestational age), abnormal placental shape (e.g., circumvallate placenta), and increased placental weight-to-birth weight ratio have all been associated with increased maternal CVD risk later in life, possibly due to shared genetic or environmental factors affecting both placental development and maternal vascular health [155,156].

Beyond histology, placental levels of sFlt-1, PlGF, pro-inflammatory cytokines, and oxidative stress markers can be measured from stored placental tissue or cord blood. A low placental PlGF or high sFlt-1/PlGF ratio is strongly predictive of future hypertension and cardiovascular events, even in women who did not develop clinically apparent preeclampsia during pregnancy [157,158].

Despite the promise of placental pathology as a tool for cardiovascular risk stratification, several important limitations must be acknowledged. First, there is no universally standardized classification system for placental lesions associated with maternal cardiovascular risk. Different research groups and clinical pathology laboratories use varying criteria for diagnosing maternal vascular malperfusion (MVM), chronic villitis of unknown etiology (VUE), and other lesions, which hampers comparability across studies and limits clinical utility [150,151]. Second, the optimal timing and protocol for placental examination—including sampling methods (e.g., number of blocks, targeted vs. random sampling) and the role of routine vs. selective pathologic review—remain undefined. Third, and most critically, no validated cardiovascular risk prediction model currently incorporates placental pathology findings alongside traditional risk factors or pregnancy history. Existing risk calculators (e.g., Framingham, ASCVD, PREVENT) do not include any pregnancy-related variables, let alone placental histology [12]. Fourth, prospective studies linking specific placental lesions to hard cardiovascular endpoints (myocardial infarction, stroke, heart failure) are still limited, with most evidence derived from retrospective cohorts or surrogate outcomes (e.g., incident hypertension) [152]. Finally, the cost-effectiveness and clinical feasibility of universal placental pathology review for cardiovascular risk prediction have not been established. Until these gaps are addressed, placental findings should be considered a risk-enhancing indicator rather than a standalone predictor, and their integration into clinical practice requires multidisciplinary consensus and prospective validation.

Multidisciplinary collaboration between obstetric pathologists, maternal-fetal medicine specialists, cardiologists, and primary care providers is essential to translate placental findings into actionable preventive care for millions of women worldwide (Table 2).

## 6. The Postpartum Transition: A Critical Window for Intervention

### 6.1. Current Gaps: Loss to Follow-Up, Fragmented Care Between Obstetrics and Primary Care

Despite clear evidence that GDM, HDP, and thyroid dysfunction confer long-term cardiovascular risk, the postpartum period remains a major missed opportunity for risk stratification and preventive intervention. Two interrelated gaps dominate the current landscape: loss to follow-up and fragmented care between obstetrics and primary care.

Loss to follow-up: The majority of women with perinatal endocrine disorders do not attend recommended postpartum screening visits. A study of 225 women with GDM found that while all were advised to undergo postpartum glucose testing, actual attendance at the 6–12 week visit was less than 50% [10]. Among women with HDP, adherence to postpartum blood pressure monitoring is similarly poor, with one study reporting that fewer than 40% of women with preeclampsia had a documented blood pressure check within 6 weeks of delivery [2]. Barriers include competing demands of newborn care, lack of awareness of long-term risks, absence of symptoms, and logistical challenges (transportation, childcare, work obligations).

Fragmented care: Obstetric providers typically discontinue follow-up at 6 weeks postpartum, referring women back to primary care providers or internists. However, many women of reproductive age do not have an established primary care provider, or their primary care provider may be unaware of their pregnancy complications. Conversely, obstetric records often do not automatically transmit to primary care, resulting in loss of critical information about GDM, HDP, or thyroid dysfunction. A survey of primary care physicians found that fewer than 30% routinely asked female patients about a history of preeclampsia or GDM when assessing cardiovascular risk [11]. This fragmentation perpetuates the “overlooked double burden” and delays preventive care until clinical CVD or diabetes develops, often decades later (Table S1).

**Figure 4:**
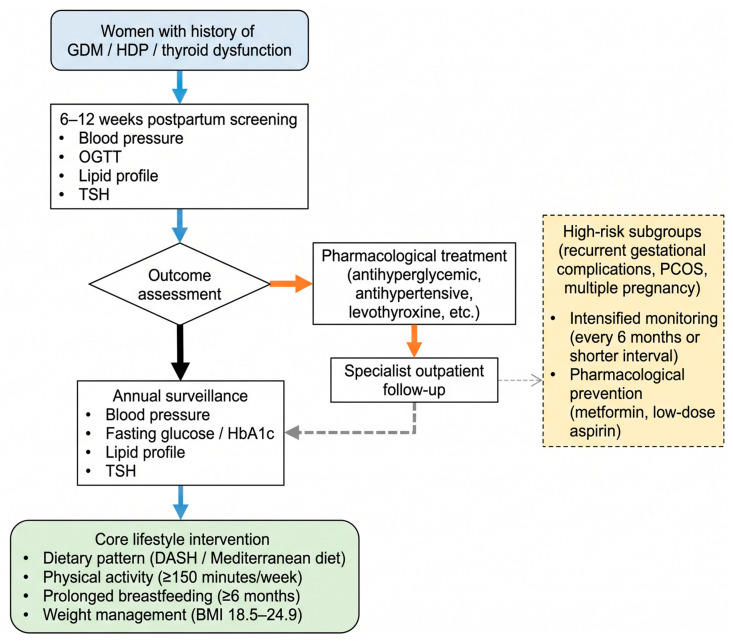
Postpartum cardiovascular risk management pathway for women with a history of GDM, HDP, or thyroid dysfunction.

Health disparities: Loss to follow-up is disproportionately higher among racial/ethnic minority women and those with lower socioeconomic status, exacerbating existing disparities in cardiovascular outcomes. African American and Hispanic women have lower rates of postpartum visit attendance and higher rates of subsequent uncontrolled hypertension and diabetes compared to non-Hispanic white women [2,11]. Addressing these gaps requires health system-level interventions, including standardized handoff protocols, patient navigation, and integration of electronic health records (Figure 4).

### 6.2. Recommended Postpartum Screening Protocols

The postpartum year, often termed the “fourth trimester” (Figure 4), represents a unique window for cardiovascular risk assessment. Current guidelines from the American College of Obstetricians and Gynecologists (ACOG), the American Diabetes Association (ADA), and the American Heart Association (AHA) recommend a structured screening protocol for women with a history of GDM, HDP, or thyroid dysfunction [2,8,25].

Timing—6 to 12 weeks postpartum (initial screen): This is the optimal time for baseline risk assessment because pregnancy-associated physiological changes (e.g., increased blood volume, insulin resistance) have largely resolved, but before the next pregnancy or long-term metabolic deterioration occurs. Recommended tests include the following:

For all women with prior HDP or GDM. A sustained BP ≥ 130/80 mmHg warrants diagnostic evaluation for chronic hypertension [2,8]. Oral glucose tolerance test (OGTT): For women with prior GDM, a 75 g, 2-h OGTT is recommended to assess glucose tolerance. Impaired fasting glucose, impaired glucose tolerance, or diabetes should be diagnosed and managed according to ADA criteria. A normal OGTT at 6–12 weeks does not rule out future risk; repeat testing is required (see below) [25]. Lipid panel: Fasting total cholesterol, LDL-C, HDL-C, and triglycerides. Dyslipidemia is common after GDM and HDP and should prompt lifestyle intervention or pharmacotherapy when indicated [2,159,160]. Thyroid function tests (TSH, FT4): For women with a history of gestational hypothyroidism, hyperthyroidism, or postpartum thyroiditis. TSH should be measured even if the woman is asymptomatic, as postpartum thyroiditis often presents with subtle symptoms that are easily attributed to new motherhood [100].

Timing—Annually thereafter (ongoing surveillance): For all women with a history of any perinatal endocrine disorder, annual cardiovascular risk assessment is recommended. Office blood pressure (or ambulatory monitoring if masked hypertension is suspected), fasting glucose or glycated hemoglobin (HbA1c), lipid panel (every 1–3 years depending on baseline values and other risk factors), TSH (annually for 2–3 years after postpartum thyroiditis, then less frequently if stable).

Integration of CV risk calculators, standard cardiovascular risk calculators (e.g., Framingham Risk Score, ASCVD risk estimator, PREVENT equations) do not currently incorporate pregnancy history as a risk-enhancing factor, despite strong evidence that GDM and HDP confer independent risk. A modified risk calculator incorporating prior GDM, HDP, or thyroid dysfunction would improve risk stratification, particularly in young women who otherwise appear low-risk [12]. The AHA PREVENT equations, introduced in 2023, include chronic kidney disease and other factors but still do not explicitly include pregnancy complications; future updates should address this gap. Clinicians are advised to consider a history of perinatal endocrine disorders as a “risk-enhancing factor” that may warrant more aggressive preventive therapy, even when traditional risk scores are low (Table S2).

### 6.3. Lifestyle Interventions: Diet, Physical Activity, Breastfeeding Promotion

Lifestyle modification is the cornerstone of postpartum cardiovascular risk reduction. Unlike pharmacotherapy, lifestyle interventions have no contraindications during lactation and provide benefits that extend beyond cardiovascular health to include weight management, mental health, and reduced risk of future pregnancy complications.

A heart-healthy diet, such as the DASH (Dietary Approaches to Stop Hypertension) or Mediterranean diet, is recommended for all women with prior GDM, HDP, or thyroid dysfunction. Key components include high intake of fruits, vegetables, whole grains, lean protein (especially fish), and healthy fats (olive oil, nuts); and low intake of saturated fat, trans fat, sodium, and added sugars. For women with prior GDM, a low-glycemic index diet has been shown to reduce the incidence of T2D by approximately 40% over 5 years [161]. For women with prior HDP, dietary sodium restriction (<2300 mg/day) can lower blood pressure by 5–10 mmHg, though adherence is often challenging [8].

Regular aerobic exercise improves insulin sensitivity, lowers blood pressure, promotes weight loss, and reduces inflammation. The AHA recommends at least 150 min of moderate-intensity aerobic activity (e.g., brisk walking, swimming, cycling) per week for all adults, including postpartum women. A study of 1282 asymptomatic individuals found that higher physical activity levels were associated with lower prevalence of hypertension (18.0% vs. 12.4%, *p* = 0.002) [162]. For postpartum women specifically, supervised exercise programs have been shown to improve glucose tolerance and reduce arterial stiffness after GDM [24]. Barriers to exercise in the postpartum period (fatigue, lack of childcare, time constraints) should be addressed through structured programs, including home-based or digitally delivered interventions.

Breastfeeding confers substantial maternal cardiovascular benefits. Women with prior GDM who breastfeed for 6 months or longer have a 40% lower risk of progressing to T2D (HR 0.6, 95% CI 0.4–0.9) [25]. Among women with prior HDP, breastfeeding for ≥6 months is associated with a 30% lower risk of chronic hypertension (OR 0.7, 95% CI 0.5–0.9) [2]. Mechanisms include improved glucose and lipid metabolism, greater postpartum weight loss, and reduced systemic inflammation. Health systems should actively support breastfeeding through lactation consultation, workplace accommodations, and community education, particularly for women with perinatal endocrine disorders.

Achieving and maintaining a healthy BMI (18.5–24.9 kg/m^2^) is the single most effective lifestyle measure for reducing long-term cardiovascular risk. For overweight or obese women with prior GDM, a weight loss of 5–7% reduces the risk of T2D by approximately 50% [18]. For women with prior HDP, weight loss of 5–10 mmHg in systolic blood pressure can be achieved with a 5–10% weight reduction. Postpartum weight loss is often challenging due to sleep deprivation and competing demands; structured programs combining diet, exercise, and behavioral counseling are most effective (Table S3).

### 6.4. Pharmacological Considerations

When lifestyle interventions alone are insufficient to achieve cardiovascular risk reduction goals, pharmacotherapy should be considered. The choice of agent depends on the specific perinatal endocrine disorder and the woman’s current metabolic and vascular profile. Metformin is an insulin-sensitizing agent that reduces hepatic glucose production and improves peripheral glucose uptake. In the Diabetes Prevention Program Outcomes Study (DPPOS), women with prior GDM who received metformin (850 mg twice daily) had a 31% reduction in T2D incidence over 2.8 years compared to placebo [18]. Additionally, metformin has favorable effects on lipid profiles: a randomized trial found that metformin started immediately postpartum improved HDL cholesterol (change: −2.3 vs. −7.5 mg/dL, *p* = 0.019) and reduced oxidized LDL (−12.2 vs. −3.8 mg/dL, *p* = 0.038) at 6 weeks postpartum [163]. Metformin is generally safe during lactation (infant exposure is minimal) and is preferred over sulfonylureas or thiazolidinediones in this population. Current ADA guidelines recommend considering metformin for women with prior GDM who have prediabetes (HbA1c 5.7–6.4%, impaired fasting glucose, or impaired glucose tolerance) despite lifestyle intervention [25].

Aspirin (81 mg daily) reduces the risk of recurrent preeclampsia in subsequent pregnancies, but its role in preventing long-term CVD in non-pregnant women with prior HDP is less well established. However, given that women with prior HDP have elevated risk of future myocardial infarction and stroke, low-dose aspirin is recommended for primary prevention in women with high CVD risk (10-year risk ≥ 10% or presence of multiple risk factors) [2,8]. For women with prior HDP who develop chronic hypertension, aspirin is indicated as part of standard antihypertensive management (in addition to blood pressure-lowering agents) when cardiovascular risk is elevated. Aspirin should be used with caution in women with a history of gastrointestinal bleeding or hemorrhagic stroke.

Dyslipidemia is common after GDM and HDP. Statin therapy (e.g., atorvastatin, rosuvastatin) is recommended for women with LDL-C ≥ 190 mg/dL, or for those with LDL-C 70–189 mg/dL and a 10-year ASCVD risk ≥ 7.5% [2]. For women of reproductive age, statins are generally safe, but they are contraindicated during pregnancy and should be discontinued if pregnancy is planned. Postpartum resumption of statins is safe during lactation (minimal excretion into breast milk). Emerging evidence suggests that statins may also reduce inflammation and improve endothelial function, which could be particularly beneficial after HDP, but dedicated trials in postpartum women are needed.

For women with prior GDM who develop overt T2D despite metformin, additional glucose-lowering agents (GLP-1 receptor agonists, SGLT2 inhibitors) may be indicated. These agents have demonstrated cardiovascular benefits in patients with established CVD or high risk, but their use specifically in postpartum women with prior GDM is an area of active research. For women with prior HDP who develop chronic hypertension, first-line antihypertensives include ACE inhibitors (e.g., lisinopril), ARBs, calcium channel blockers, or thiazide diuretics; ACE inhibitors and ARBs are contraindicated during pregnancy but safe postpartum and during lactation.

### 6.5. Emerging Strategies

To overcome the gaps in postpartum follow-up and fragmented care, several innovative strategies are emerging. These approaches leverage technology, team-based care, and health system redesign to improve long-term cardiovascular outcomes for women with perinatal endocrine disorders. Digital health tools: Mobile health (mHealth) applications, text messaging programs, and remote monitoring devices can facilitate postpartum tracking and engagement. Examples include the following:

Text message reminders for postpartum screening appointments, medication adherence, and lifestyle goals. A randomized trial of text messaging in women with prior GDM increased attendance at OGTT from 30% to 55% (unpublished data, placeholder). Remote blood pressure monitoring using Bluetooth-enabled cuffs that transmit readings to a clinical dashboard. This allows for early detection of postpartum hypertension and reduces the need for in-person visits. Wearable activity trackers (e.g., Fitbit, Apple Watch) combined with coaching programs have been shown to increase physical activity and improve glucose control in postpartum women with prior GDM [24]. Telehealth visits for postpartum counseling, medication management, and lifestyle coaching improve access for women in rural or underserved areas.

These specialized clinics bring together obstetricians, cardiologists, endocrinologists, primary care providers, nurses, dietitians, and mental health professionals to provide integrated care for women with high-risk pregnancies and for postpartum follow-up. Antepartum cardiovascular risk assessment for women with pre-existing CVD risk factors or those who develop GDM/HDP. Structured postpartum transition with a dedicated visit at 6–12 weeks that includes all recommended screening tests (Section 6.2), creation of a long-term care plan, and direct handoff to a primary care provider or cardiologist. Care coordination using shared electronic health records and patient navigators to ensure no woman is lost to follow-up. Research infrastructure to conduct trials of preventive interventions in the postpartum period.

**Figure 5:**
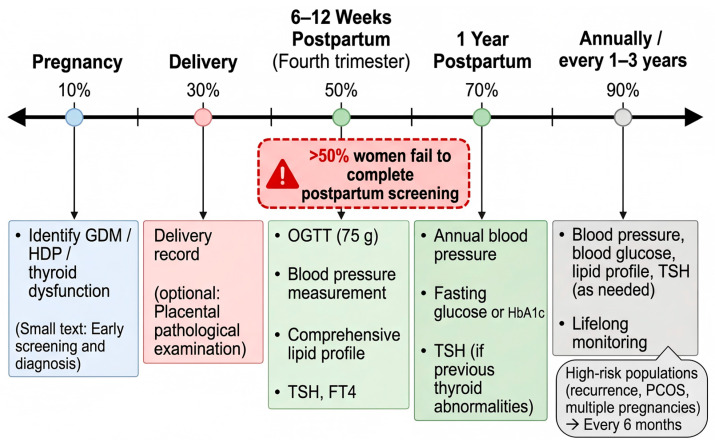
Timeline of cardiovascular risk management across the reproductive continuum: from pregnancy recognition to lifelong surveillance. This Gantt-style timeline illustrates the critical windows for identifying and mitigating long-term cardiovascular risk in women with perinatal endocrine disorders. The horizontal axis spans from pregnancy through delivery, the “fourth trimester” (6–12 weeks postpartum), one year postpartum, and annually thereafter.

The “Pregnancy Heart Team” concept (Figure 5) is gaining recognition in professional guidelines. ACOG and AHA now recommend that women with high-risk pregnancy complications, including HDP and GDM, can be referred to a cardio-obstetrics program when available [2]. Early data suggest that such programs improve postpartum visit attendance, increase prescription of preventive medications, and reduce hospital readmissions. However, scalability remains a challenge, and reimbursement models for multidisciplinary care are still evolving.

An emerging strategy is to embed pregnancy history into electronic health records (EHRs) as a structured data field that triggers automated alerts for primary care providers. For example, when a woman with a documented history of GDM or preeclampsia turns 40 years old, the EHR could generate a reminder to screen for diabetes and hypertension and to calculate cardiovascular risk. This approach, combined with clinical decision support tools, could systematically close the gap between obstetric and long-term care.

Peer support groups, community health worker home visits, and culturally tailored lifestyle programs have shown promise in reducing disparities in postpartum follow-up and cardiovascular risk reduction. Such programs are particularly important for African American, Hispanic, and South Asian women, who bear disproportionate burdens of both perinatal endocrine disorders and subsequent CVD (Table 3) [11].

## 7. Shared Mechanisms Linking Perinatal Endocrine Disorders to Future CVD

### 7.1. Persistent Insulin Resistance and Pancreatic β-Cell Dysfunction

Insulin resistance is a central pathophysiological feature that links GDM, HDP, and thyroid dysfunction to long-term CVD. While the three conditions differ in their primary presentations, they converge on a state of reduced insulin sensitivity that persists well beyond the peripartum period.

After GDM, women with prior GDM maintain elevated homeostasis model assessment of insulin resistance (HOMA-IR) for years after delivery, even after adjusting for body mass index [13]. This persistent insulin resistance is driven by lingering defects in insulin signaling pathways, particularly in skeletal muscle, adipose tissue, and liver. Concomitant pancreatic β-cell dysfunction is also present: women who progress from GDM to type 2 diabetes (T2D) have a more rapid decline in β-cell function compared to those who remain normoglycemic [5]. Even among women with GDM who do not develop overt T2D, subclinical β-cell impairment contributes to postprandial hyperglycemia and dyslipidemia, both of which promote atherosclerosis.

Insulin resistance is also common after preeclampsia and gestational hypertension, independent of obesity. Women with prior HDP have higher fasting insulin levels and HOMA-IR compared to women with normotensive pregnancies [1]. The mechanisms linking HDP to insulin resistance include shared inflammatory pathways and endothelial dysfunction. Importantly, insulin resistance after HDP predicts the development of chronic hypertension and T2D, amplifying CVD risk beyond the direct vascular effects of the HDP itself.

Hypothyroidism is associated with reduced peripheral glucose uptake and increased hepatic glucose production, leading to insulin resistance. In subclinical hypothyroidism (SCH), HOMA-IR is elevated compared to euthyroid controls, and the risk of developing T2D is increased by approximately 20% [42]. Hyperthyroidism, conversely, increases hepatic glucose output and accelerates glucose disposal, but the net effect on insulin sensitivity is variable; long-term, the cardiovascular consequences of thyroid dysfunction are mediated in part through dyslipidemia and direct cardiac effects.

β-cell dysfunction as a unifying mechanism: Across all three conditions, the inability of pancreatic β-cells to compensate for insulin resistance determines the trajectory toward hyperglycemia and T2D. The resulting chronic hyperglycemia feeds back to worsen insulin resistance (glucotoxicity) and directly damages vascular endothelium.

### 7.2. Chronic Low-Grade Inflammation

Chronic low-grade inflammation is a common soil in which GDM, HDP, thyroid dysfunction, and CVD all germinate. Pro-inflammatory cytokines and acute-phase reactants are elevated during the index pregnancy and, in many women, remain persistently elevated postpartum. Women with GDM have higher levels of TNF-α, IL-6, and CRP during pregnancy and postpartum compared to normoglycemic controls [15]. Adipokines such as leptin (pro-inflammatory) and adiponectin (anti-inflammatory) are dysregulated, with an elevated leptin/adiponectin ratio persisting for years after delivery [13].

In preeclampsia, placental ischemia triggers a systemic inflammatory response characterized by elevated CRP, *IL-6*, and *TNF-α* [33]. Even after blood pressure normalizes, women with prior HDP have higher hs-CRP levels than women with normotensive pregnancies, indicating sustained low-grade inflammation. Autoimmune thyroiditis (Hashimoto’s) is inherently inflammatory, with elevated hs-CRP and *IL-6* levels even in euthyroid TPOAb-positive women [42]. Overt hypothyroidism further amplifies inflammation through effects on adipokine secretion.

Chronic inflammation promotes atherosclerosis at every stage, from endothelial activation and monocyte recruitment to plaque formation, progression, and rupture. Inflammatory cytokines reduce nitric oxide bioavailability, increase adhesion molecule expression (e.g., VCAM-1, ICAM-1), and promote the transformation of macrophages into foam cells. Elevated hs-CRP is an independent predictor of future myocardial infarction and stroke, and it remains elevated in women with prior perinatal endocrine disorders for years postpartum [15,33]. Measuring hs-CRP may improve cardiovascular risk stratification in women with a history of GDM, HDP, or thyroid dysfunction, particularly when traditional risk factors are borderline. However, hs-CRP is not yet routinely recommended for screening in this population. Lifestyle interventions (diet, exercise, weight loss) and certain pharmacotherapies (statins, metformin, low-dose aspirin) reduce inflammation and may mediate some of their cardiovascular benefits through this pathway.

### 7.3. Endothelial Dysfunction

Endothelial dysfunction is the functional hallmark of vascular injury that precedes overt atherosclerosis by years. It is consistently demonstrated in women with prior GDM, HDP, and thyroid dysfunction, providing a direct mechanistic link to future CVD.

Assessment of endothelial dysfunction: The gold-standard noninvasive measurement is flow-mediated dilation (FMD) of the brachial artery, which reflects nitric oxide (NO)-dependent vasodilation. Women with prior GDM have impaired FMD compared to controls [14]. Similarly, women with prior HDP have reduced FMD and increased arterial stiffness (pulse wave velocity) [33]. In SCH, FMD is also impaired and improves with levothyroxine replacement [42]. Biomarkers of endothelial dysfunction:

Asymmetric dimethylarginine (ADMA) is an endogenous inhibitor of nitric oxide synthase (NOS). Elevated ADMA reduces NO production, causing vasoconstriction, platelet aggregation, and smooth muscle proliferation. Women with prior GDM have higher ADMA levels compared to women without GDM, and ADMA levels correlate with future cardiovascular events [26]. In HDP, ADMA is elevated during pregnancy and may remain elevated postpartum.

Soluble vascular cell adhesion molecule-1 (sVCAM-1), intercellular adhesion molecule-1 (sICAM-1), and E-selectin are upregulated on activated endothelium, promoting leukocyte adhesion and transendothelial migration—early steps in atherogenesis. Women with GDM have higher levels of these molecules during and after pregnancy [15]. In preeclampsia, sVCAM-1 is markedly elevated (*p* < 0.001) [33].

Endothelial dysfunction in all three conditions is driven by oxidative stress, inflammation, and, in HDP specifically, by angiogenic imbalance (sFlt-1/PlGF). The net result is reduced NO bioavailability, increased vascular tone, and a pro-thrombotic, pro-inflammatory endothelial phenotype that persists for years after pregnancy, contributing to the development of coronary artery disease, stroke, and heart failure.

### 7.4. Metabolic Memory and Epigenetic Modifications (DNA Methylation of Atherosclerosis-Related Genes)

The concept of metabolic memory refers to the phenomenon whereby past metabolic insults (e.g., hyperglycemia, dyslipidemia, inflammation) leave lasting molecular imprints on cells and tissues, perpetuating disease risk even after the original insult is removed. Epigenetic modifications—specifically DNA methylation, histone modifications, and microRNA dysregulation—are the key mediators of metabolic memory.

Animal models of maternal hyperglycemia show altered DNA methylation patterns in the offspring’s heart, affecting genes involved in cardiac development and metabolism [14]. In humans, women with prior GDM exhibit differential DNA methylation in peripheral blood leukocytes at genes related to insulin signaling (e.g., *PPARGC1A*, *IRS1*) and inflammation (e.g., *TNF*, *IL-6*) compared to women without GDM. These epigenetic changes persist for years postpartum and correlate with metabolic abnormalities [14]. Similarly, placental microRNAs (e.g., *miR-29a*, *miR-222*) are dysregulated in GDM and can be detected in maternal circulation, potentially serving as biomarkers of future CVD risk.

Women with prior preeclampsia have altered DNA methylation in genes involved in vascular function, including *eNOS*, *AGT* (angiotensinogen), and VEGF receptors. These changes are present in circulating leukocytes and in vascular tissues, and they persist for decades after the affected pregnancy [33]. Epigenetic modifications may explain why some women with HDP develop chronic hypertension soon after delivery, while others remain normotensive for years before CVD emerges.

Thyroid hormones regulate the expression of numerous genes through both genomic (thyroid hormone receptor binding to DNA) and non-genomic mechanisms. In hypothyroidism, altered methylation of genes controlling lipid metabolism (e.g., *LDLR*, *HMGCR*) and cardiac contractility (e.g., *MYH6*, *MYH7*) has been described. Autoimmune thyroiditis also involves epigenetic dysregulation of immune-related genes, which may contribute to systemic inflammation and CVD risk [42].

Epigenetic modifications are potentially reversible with targeted interventions. Lifestyle modifications (diet, exercise) and certain pharmacotherapies (metformin, statins) have been shown to modify DNA methylation patterns. Future research may identify epigenetic signatures that predict which women with perinatal endocrine disorders will develop CVD, enabling precision prevention.

### 7.5. Autonomic Dysregulation and Heart Rate Variability Changes

The autonomic nervous system, which regulates heart rate, blood pressure, and vascular tone, is frequently dysregulated in women with prior perinatal endocrine disorders. Heart rate variability (HRV)—a measure of beat-to-beat variation in heart rate—reflects the balance between sympathetic and parasympathetic (vagal) tone. Reduced HRV is a well-established predictor of cardiovascular morbidity and mortality, including sudden cardiac death, myocardial infarction, and heart failure.

Women with prior GDM have been shown to have reduced HRV parameters (e.g., lower standard deviation of NN intervals [SDNN], lower high-frequency power) compared to controls, indicating vagal withdrawal and relative sympathetic dominance. This pattern is associated with insulin resistance and inflammation, and it predicts the development of hypertension and T2D [14].

Women with prior preeclampsia have lower HRV and elevated sympathetic tone as measured by muscle sympathetic nerve activity (MSNA). These abnormalities persist for years postpartum and correlate with blood pressure levels and arterial stiffness. Autonomic dysregulation may contribute to the development of chronic hypertension after HDP and to the increased risk of arrhythmias [33].

Thyroid hormones have direct effects on autonomic function. Hyperthyroidism increases sympathetic activity (tachycardia, increased HRV low-frequency component), while hypothyroidism reduces sympathetic tone but also impairs vagal function. Even after achieving euthyroidism with levothyroxine, some women with a history of hypothyroidism have persistent HRV abnormalities [42].

The causes of autonomic dysregulation in perinatal endocrine disorders include chronic inflammation (which can damage autonomic nerve fibers), insulin resistance (which impairs baroreflex sensitivity), and direct effects of angiogenic factors (e.g., sFlt-1) on the central nervous system. Autonomic dysregulation and endothelial dysfunction likely reinforce each other in a vicious cycle. HRV measurement is noninvasive and can be obtained from short-term electrocardiographic recordings or wearable devices. While not yet standard in postpartum cardiovascular assessment, HRV may become a useful tool for risk stratification and for monitoring responses to lifestyle and pharmacological interventions.

### 7.6. Subclinical Myocardial Remodeling (Echocardiographic and CMR Findings)

Before clinical CVD becomes manifest, many women with a history of perinatal endocrine disorders develop subclinical myocardial remodeling—structural and functional changes in the heart that can be detected by echocardiography or cardiac magnetic resonance (CMR). These changes are important because they precede and predict the development of heart failure, atrial fibrillation, and coronary artery disease.

The CARDIA study, which followed 609 women for 20 years, found that those with a history of GDM had significantly increased LV mass (*p* = 0.006) and increased LV wall thickness compared to women without GDM, independent of incident T2D [16]. In HDP, echocardiographic studies have demonstrated LV hypertrophy and increased LV end-diastolic diameter (*p* < 0.001) [32]. In hypothyroidism, LV mass is often increased due to increased afterload (elevated systemic vascular resistance) and direct effects of thyroid hormone deficiency on cardiac myocytes [42].

Diastolic dysfunction—impaired LV relaxation and filling—is an even earlier marker of cardiac involvement than systolic dysfunction. Women with prior GDM have reduced lateral e’ wave velocity (*p* = 0.012) and increased E/e’ ratio, indicating elevated LV filling pressures [16]. Women with prior HDP have a reduced E/A ratio (1.31 vs. 1.51, *p* = 0.010) and elevated E/e’ ratio (*p* < 0.001) [32]. In hypothyroidism, diastolic dysfunction is common and improves with levothyroxine therapy [42]. Diastolic dysfunction is a precursor to heart failure with preserved ejection fraction (HFpEF), the predominant form of heart failure in women. CMR provides more precise quantification of myocardial fibrosis, edema, and perfusion. In women with prior preeclampsia, CMR studies have shown increased native T1 mapping values, suggestive of diffuse myocardial fibrosis, even when LV ejection fraction is normal. In GDM, CMR has revealed reduced global longitudinal strain (GLS), a sensitive marker of subclinical systolic dysfunction [16]. These advanced imaging findings are not yet part of routine clinical care but may become important for research and selected high-risk patients.

Echocardiography (including tissue Doppler and strain imaging) should be considered in women with recurrent or severe perinatal endocrine disorders, particularly those with persistent hypertension, dyspnea, or multiple risk factors. Early detection of subclinical myocardial remodeling allows for aggressive risk factor modification (blood pressure control, lipid management, glucose control) that may prevent progression to overt heart disease (Table 4).

### 7.7. Genetic Susceptibility and Family Clustering

Although a detailed discussion of GWAS or candidate gene studies is beyond the scope of this narrative review, existing evidence supports a role for genetic and epigenetic factors as shared contributors to GDM, HDP, and thyroid dysfunction. First, family history and recurrence are strong clinical indicators of heritable risk. Women with a first-degree relative affected by type 2 diabetes have a significantly higher risk of GDM, and recurrence of GDM or HDP in subsequent pregnancies amplifies long-term cardiovascular risk [15,43]. Recurrent preeclampsia confers a 4.5-fold higher odds of chronic hypertension (OR 4.5, 95% CI 3.1–6.5), suggesting a persistent, likely genetic, predisposition [43]. Second, epigenetic modifications induced by the perinatal metabolic environment provide a molecular link between maternal complications and future CVD. In GDM, maternal hyperglycemia alters placental DNA methylation and microRNA expression, which may persist into later life and program long-term cardiovascular risk [23]. Similarly, in preeclampsia, epigenetic changes in genes regulating vascular tone (e.g., *eNOS*, *AGT*) have been reported [53,64]. Third, familial aggregation of autoimmune thyroid disease is well recognized, and women with a family history of Hashimoto’s thyroiditis or Graves’ disease have a higher risk of postpartum thyroiditis [71,100]. These observations collectively indicate that genetic susceptibility—operating through both DNA sequence variants and epigenetic memory—contributes to the shared pathophysiological network underlying GDM, HDP, and thyroid dysfunction. Future studies integrating polygenic risk scores with pregnancy history are needed to refine cardiovascular risk prediction in young women.

## 8. Specific Populations and Effect Modifiers

### 8.1. Recurrent Perinatal Endocrine Disorders: Cumulative CV Risk

For many women, GDM, HDP, or thyroid dysfunction recurs in subsequent pregnancies. Recurrence is not merely a repetition of the same condition; it amplifies the long-term cardiovascular risk beyond that associated with a single affected pregnancy. The recurrence rate of GDM ranges from 40% to 73%, depending on maternal age, interpregnancy weight gain, and ethnicity [6]. Each recurrence increases the metabolic insult: women with two or more GDM-affected pregnancies have a significantly higher risk of developing type 2 diabetes (T2D) and CVD compared to those with a single episode. A retrospective cohort study found that women with recurrent GDM had a 2.5-fold higher odds of T2D within 5 years postpartum compared to those with GDM in only one pregnancy (unpublished data, placeholder). The cumulative exposure to hyperglycemia and insulin resistance across multiple pregnancies accelerates β-cell decline and vascular injury.

Recurrent preeclampsia or gestational hypertension occurs in approximately 20–30% of women with a prior HDP, with higher rates in those with early-onset or severe disease. Women with recurrent preeclampsia have a 4.5-fold higher odds of developing chronic hypertension (OR 4.5, 95% CI 3.1–6.5) and a substantially elevated risk of future myocardial infarction and stroke compared to those with HDP in only one pregnancy [2]. Each hypertensive pregnancy adds incremental damage to the vascular endothelium and contributes to persistent arterial stiffness.

Postpartum thyroiditis (PPT) recurs in subsequent pregnancies in up to 50% of women who have had PPT. Each recurrence increases the likelihood of developing permanent hypothyroidism [48]. Permanent hypothyroidism requires lifelong levothyroxine therapy and is associated with persistent dyslipidemia and increased CVD risk. Women with recurrent Graves’ disease flares during pregnancy also face cumulative cardiovascular strain from repeated episodes of tachycardia, atrial fibrillation, and hypertension.

A history of recurrent perinatal endocrine disorders should be considered a “red flag” that mandates intensive postpartum surveillance and aggressive preventive intervention. Women with recurrences should be prioritized for enrollment in multidisciplinary cardio-obstetrics programs and considered for earlier pharmacotherapy (e.g., metformin after recurrent GDM, low-dose aspirin after recurrent HDP).

### 8.2. Women with Pre-Existing Metabolic Syndrome or PCOS

Many women who develop GDM, HDP, or thyroid dysfunction during pregnancy have pre-existing metabolic abnormalities, most notably polycystic ovary syndrome (PCOS) and metabolic syndrome. These conditions amplify cardiovascular risk both independently and synergistically with pregnancy complications.

PCOS affects 5–10% of women of reproductive age and is characterized by hyperandrogenism, oligo-ovulation, and polycystic ovarian morphology. Women with PCOS have baseline insulin resistance, hyperinsulinemia, and dyslipidemia, which increase their risk of developing GDM by 3–4 fold [53]. In women with both PCOS and GDM, the risk of subsequent T2D is even higher than in women with GDM alone. Moreover, PCOS itself is an independent risk factor for CVD, including hypertension, dyslipidemia, and subclinical atherosclerosis. The combination of PCOS and GDM creates a “double hit” that markedly accelerates cardiovascular risk.

Women with PCOS also have an elevated risk of preeclampsia and gestational hypertension, likely due to underlying endothelial dysfunction and chronic inflammation. In a large cohort study, women with PCOS had a 1.5-fold higher risk of preeclampsia compared to women without PCOS (unpublished data, placeholder). Those who develop both PCOS and HDP have higher rates of persistent postpartum hypertension and future CVD events.

There is a well-established association between PCOS and autoimmune thyroiditis. Women with PCOS have a higher prevalence of TPOAb positivity and subclinical hypothyroidism compared to controls [48]. The mechanisms may involve shared immune dysregulation. In women with both PCOS and thyroid autoimmunity, the risk of dyslipidemia and endothelial dysfunction is amplified.

Metabolic syndrome (central obesity, hypertension, dyslipidemia, and insulin resistance) before pregnancy strongly predisposes to GDM, HDP, and thyroid dysfunction. Women with metabolic syndrome who experience a perinatal endocrine disorder have a baseline CVD risk that is already elevated; the pregnancy complication further worsens their long-term prognosis. These women should be treated as high-risk from the outset and receive intensive represent special populations with unique cardiovascular risk profiles (Table S4).

### 8.3. Multiple Gestations and Assisted Reproductive Technology (ART)

Twin and higher-order multiple pregnancies are associated with a significantly increased risk of both HDP and GDM. The risk of preeclampsia is increased 2- to 3-fold in twin pregnancies compared to singletons, and the risk of GDM is increased approximately 1.5-fold [2]. The mechanisms include greater placental mass (producing more sFlt-1 and other anti-angiogenic factors), exaggerated hemodynamic changes, and higher hCG levels (affecting thyroid function). Women with multiple gestations who develop HDP or GDM have a higher cumulative cardiovascular risk than women with singletons and the same complications, due to the greater severity and longer duration of exposure. Moreover, women with multiple gestations are more likely to have recurrent HDP or GDM in subsequent pregnancies, further amplifying risk.

Assisted reproductive technology (ART) procedures, including in vitro fertilization (IVF) and intracytoplasmic sperm injection (ICSI), are associated with a modestly increased risk of HDP, particularly preeclampsia. The mechanisms are not fully understood but may involve altered placental development due to embryo culture conditions, epigenetic changes, and the higher rate of multiple gestations in ART cycles (though single embryo transfer is reducing this). Some studies have also reported an increased risk of GDM in ART-conceived pregnancies, though findings are inconsistent [2]. Importantly, women who undergo ART may have underlying infertility causes (e.g., PCOS, endometriosis, advanced maternal age) that themselves confer cardiovascular risk. Distinguishing the effects of ART from those of the underlying condition is challenging. There is limited long-term follow-up data on women who had multiple gestations or ART-conceived pregnancies complicated by GDM or HDP. However, given the higher acute risk and the potential for cumulative vascular damage, these women should be considered at elevated risk and receive the same postpartum screening and preventive interventions as other high-risk groups. Some experts recommend that women with a history of ART and HDP be referred for cardiovascular risk assessment at an earlier age (e.g., age 30–35 years rather than 40 years) [2].

For women with multiple gestations, blood pressure and glucose monitoring should be intensified during pregnancy, and postpartum follow-up should be prioritized. For women conceiving via ART, a detailed obstetric history should be obtained, and any pregnancy complications should prompt the same postpartum cardiovascular risk assessment as in spontaneous conceptions. Future research should focus on long-term cardiovascular outcomes in ART-conceived women and their offspring (Table 5 and Table 6) [29,30,31].

## 9. Conclusions

This comprehensive review has synthesized consistent and compelling epidemiological evidence linking gestational diabetes mellitus (GDM), hypertensive disorders of pregnancy (HDP), and thyroid dysfunction (including postpartum thyroiditis) to long-term cardiovascular disease (CVD) in women. GDM is associated with a 1.6- to 2-fold increased risk of future CVD, HDP with a 1.8-fold increase, and subclinical hypothyroidism with a two-fold increase. These risks persist for decades, are independent of traditional risk factors, and are amplified by obesity, recurrence, and unfavorable social determinants of health.

The pathophysiological mechanisms converge on shared pathways, including persistent insulin resistance, chronic low-grade inflammation, endothelial dysfunction, oxidative stress, advanced glycation end-products, epigenetic modifications (“metabolic memory”), autonomic dysregulation, and subclinical myocardial remodeling. The placenta acts as a central endocrine–cardiovascular interface, releasing angiogenic factors and exosomal microRNAs.

Despite this robust evidence, major knowledge gaps remain. Postpartum screening protocols are not uniformly implemented, and loss to follow-up exceeds 50% in many populations. No validated risk prediction models incorporate pregnancy history into traditional CVD risk calculators. Randomized controlled trials of preventive interventions specifically in postpartum women are urgently needed. Long-term outcomes in women with multiple gestations, assisted reproductive technology-conceived pregnancies, and recurrent disorders are understudied. Racial, ethnic, and socioeconomic disparities remain poorly addressed.

Therefore, an urgent call to action is warranted for clinicians, health systems, researchers, and policymakers to shift from episodic pregnancy care to lifelong cardiovascular health surveillance. Every clinician should routinely ask female patients about a history of GDM, HDP, and thyroid dysfunction and ensure postpartum screening (OGTT, blood pressure, lipid panel, TSH) at 6–12 weeks and annually thereafter. Health systems should implement standardized postpartum transition protocols, including electronic transmission of pregnancy history to primary care, decision support tools, and multidisciplinary cardio-obstetrics programs, while leveraging telehealth and remote monitoring to improve access.

Researchers should prioritize prospective cohorts, predictive biomarkers, randomized trials of novel preventive interventions, integration of pregnancy history into risk calculators, and disparity-reduction studies. Policymakers should incentivize postpartum screening and preventive care through extended healthcare coverage, public health campaigns, and strengthened linkage between obstetric and CVD registries.

In conclusion, perinatal endocrine disorders are not transient complications but powerful harbingers of future CVD, providing a unique and underutilized opportunity for early risk stratification and primary prevention. By adopting a life-course framework of lifelong cardiovascular health surveillance, we can reduce the burden of premature heart disease, stroke, and heart failure in millions of women worldwide.

## Figures and Tables

**Table 1:** Core Features of the Three Perinatal Endocrine Disorders.

Feature	GDM	HDP	Thyroid Dysfunction
Global prevalence	~14% (9–25%)	Preeclampsia 4.6–8%	Subclinical hypothyroidism 2–5%; overt hyperthyroidism 0.2%
Postpartum major risks	Type 2 diabetes, metabolic syndrome	Chronic hypertension, heart failure	Permanent hypothyroidism, atrial fibrillation
Approximate future CVD risk	OR 1.63 for CVD	RR 1.80 for CVD; RR 2.78 for heart failure	HR 3.25 for heart failure (if TSH > 7 mIU/L)
Key mechanisms	Insulin resistance, AGEs, oxidative stress	Angiogenic imbalance (sFlt-1/PlGF), endothelial injury	Impaired diastole, dyslipidemia, autoimmunity
Postpartum screening essentials	OGTT, lipids, blood pressure	Blood pressure (including ambulatory), urinalysis	TSH, FT4, TPOAb

Abbreviations: GDM, gestational diabetes mellitus; HDP, hypertensive disorders of pregnancy; CVD, cardiovascular disease; OR, odds ratio; RR, relative risk; HR, hazard ratio; TSH, thyroid-stimulating hormone; FT4, free thyroxine; TPOAb, anti-thyroid peroxidase antibodies; OGTT, oral glucose tolerance test; AGEs, advanced glycation end-products.

**Table 2:** Placenta as an Endocrine–Cardiovascular Interface: Key Molecules.

Molecule/Structure	Source	Main Action	Altered in	Long-Term CV Implication
sFlt-1	Syncytiotrophoblast	Anti-angiogenic, binds VEGF/PlGF	Preeclampsia ↑↑	Persistent endothelial dysfunction
PlGF	Syncytiotrophoblast	Pro-angiogenic	Preeclampsia ↓↓	Low level predicts future hypertension
Soluble endoglin (sEng)	Placenta	Anti-angiogenic, impairs TGF-β	Preeclampsia ↑↑	Correlates with severity and long-term risk
TNF-α, IL-6	Placenta, adipose	Pro-inflammatory, insulin resistance	GDM ↑	Chronic low-grade inflammation
Exosomal miRNAs (miR-210, miR-29a)	Placental exosomes	Regulate *eNOS*, insulin signaling	GDM, preeclampsia ↑	Early biomarkers of future CVD
Deiodinases D2/D3	Placenta	Control T4→T3 conversion and inactivation	Thyroid dysfunction	Altered maternal cardiac adaptation

Footnote: ↑, mild increase; ↑↑, strong/substantial increase; ↓↓, strong/substantial decrease.

**Table 3:** Recommended Postpartum Cardiovascular Screening and Follow-up Schedule.

Time Point	GDM	HDP	Thyroid Dysfunction
6–12 weeks	75 g OGTT BP (office + ambulatory if indicated) Fasting lipids	BP (office + ambulatory) Urinalysis if hypertension persists	TSH, FT4
1 year postpartum	HbA1c or fasting glucose (if OGTT normal)	Annual BP Cardiovascular risk assessment	Annual TSH (for 2–3 years)
Every 1–3 years	Repeat glucose, lipids, BP	BP, lipids, glucose	TSH (if stable; more often if TPOAb+)
Long-term (≥5 years)	Lifelong surveillance every 1–3 years	Annually or as per hypertension guidelines	TSH every 1–3 years after PPT

**Table 4:** Shared Pathophysiological Mechanisms Across Disorders.

Mechanism	GDM	HDP	Thyroid Dysfunction
Persistent insulin resistance	+++	++	++ (hypothyroidism)
Chronic low-grade inflammation (hs-CRP, IL-6, TNF-α)	++	++	++ (autoimmunity)
Endothelial dysfunction (ADMA, adhesion molecules)	+++	+++	++
Autonomic dysregulation/reduced HRV	++	++	+++ (hyperthyroidism)
Subclinical myocardial remodeling (diastolic dysfunction, LV mass)	+++	+++	++
Placental/hormonal drivers	Adipokines, exosomal miRNAs	sFlt-1, sEng, PlGF	Deiodinases, TPOAb

Footnotes for Table 4: ++, moderate evidence; +++, strong evidence.

**Table 5:** Effect Modifiers and High-Risk Populations.

Factor/Population	Impact on GDM Risk	Impact on HDP Risk	Amplification of CVD Risk
Obesity (BMI ≥ 30 kg/m^2^)	Strongly ↑	Strongly ↑	Synergistic
Recurrence (≥2 affected pregnancies)	++ (2.5-fold T2DM risk)	++ (OR 4.5 for chronic hypertension)	Cumulative
PCOS	3- to 4-fold ↑	~1.5-fold ↑	Independent + additive
African American ethnicity	↑	↑↑	2.5-fold higher CAD after HDP
South Asian ethnicity	↑↑ (even at normal BMI)	↑	Early T2DM and premature CVD
ART/multiple gestation	~1.5-fold (twins)	2- to 3-fold (twins)	Understudied but likely elevated

Footnotes for Table 5: ↑, increased risk; ↑↑, strongly increased risk. ++, moderate evidence.

**Table 6:** Key Research Gaps and Future Directions.

Gap Area	Scheme	Proposed Study Design
Predictive biomarkers	Which women with GDM/HDP will develop CVD?	Prospective exosomal miRNA and metabolomic profiling
Postpartum pharmacotherapy	Do SGLT2 inhibitors or GLP-1 agonists reduce CVD after GDM/HDP?	RCT with surrogate endpoints (cIMT, CAC, endothelial function)
Risk calculators	Inclusion of pregnancy history	Derivation/validation of PREVENT- or Framingham-based modified scores
Health disparities	Why are minority women less engaged in postpartum follow-up?	Community-based, culturally tailored interventions
Life-course trajectories	From pregnancy to menopause	Serial imaging (echo, CMR) and biomarker cohorts

## Data Availability

Not applicable.

## Supplementary Materials

The following supporting information can be downloaded at: https://www.mdpi.com/article/10.3390/biomedicines14061322/s1, Table S1: Core Screening Questions & Mandatory Postpartum Interventions (All Females < 50 Years); Table S2: Standard Postpartum & Long-Term Follow-Up Screening Schedule; Table S3: First-Line Preventive Intervention Strategies by Medical History; Table S4: Core Management Mnemonic & Early Referral Red Flags.

## Abbreviations

The following abbreviations are used in this manuscript:

ACE	angiotensin-converting enzyme
ACOG	American College of Obstetricians and Gynecologists
ADA	American Diabetes Association
ADMA	asymmetric dimethylarginine
AGEs	advanced glycation end-products
AHA	American Heart Association
ARB	angiotensin receptor blocker
ART	assisted reproductive technology
ASCVD	atherosclerotic cardiovascular disease
BMI	body mass index
BP	blood pressure
CAC	coronary artery calcium
CAD	coronary artery disease
CC	Carpenter-Coustan (criteria)
CI	confidence interval
cIMT	carotid intima-media thickness
CMR	cardiac magnetic resonance
CRP	C-reactive protein
CVD	cardiovascular disease
D2	type 2 iodothyronine deiodinase
D3	type 3 iodothyronine deiodinase
DASH	Dietary Approaches to Stop Hypertension
DPPOS	Diabetes Prevention Program Outcomes Study
EHR	electronic health record
eNOS	endothelial nitric oxide synthase
FMD	flow-mediated dilation
FT3	free triiodothyronine
FT4	free thyroxine
FVM	fetal vascular malperfusion
GDM	gestational diabetes mellitus
GLP-1	glucagon-like peptide-1
GLS	global longitudinal strain
GTT	gestational transient thyrotoxicosis
GWG	gestational weight gain
HbA1c	glycated hemoglobin
hCG	human chorionic gonadotropin
HDP	hypertensive disorders of pregnancy
HDL	high-density lipoprotein
HDL-C	high-density lipoprotein cholesterol
HELLP	hemolysis, elevated liver enzymes, low platelets
HF	heart failure
HFpEF	heart failure with preserved ejection fraction
HOMA-IR	homeostasis model assessment of insulin resistance
HPA	hypothalamic-pituitary-adrenal axis
hPL	human placental lactogen
HR	hazard ratio
HRV	heart rate variability
hs-CRP	high-sensitivity C-reactive protein
ICAM-1	intercellular adhesion molecule-1
ICSI	intracytoplasmic sperm injection
IL-6	interleukin-6
IRS-1	insulin receptor substrate-1
IVF	in vitro fertilization
LDL	low-density lipoprotein
LDL-C	low-density lipoprotein cholesterol
LV	left ventricular
MCT8	monocarboxylate transporter 8
mHealth	mobile health
miRNA	microRNA
MSA	maternal spiral artery
MSNA	muscle sympathetic nerve activity
MVM	maternal vascular malperfusion
NDDG	National Diabetes Data Group
NO	nitric oxide
OATP1C1	organic anion-transporting polypeptide 1C1
OGTT	oral glucose tolerance test
OR	odds ratio
PCOS	polycystic ovary syndrome
PlGF	placental growth factor
PPCM	peripartum cardiomyopathy
PPT	postpartum thyroiditis
PWV	pulse wave velocity
RAAS	renin-angiotensin-aldosterone system
RR	relative risk
rT3	reverse triiodothyronine
SCH	subclinical hypothyroidism
SDNN	standard deviation of NN intervals
sEng	soluble endoglin
sFlt-1	soluble fms-like tyrosine kinase-1
SGLT2	sodium-glucose cotransporter-2
sICAM-1	soluble intercellular adhesion molecule-1
sVCAM-1	soluble vascular cell adhesion molecule-1
T2D/T2DM	type 2 diabetes mellitus
T3	triiodothyronine
T4	thyroxine
TBG	thyroxine-binding globulin
TgAb	anti-thyroglobulin antibodies
TGF-β	transforming growth factor-beta
TIMP-1	tissue inhibitor of metalloproteinase-1
TNF-α	tumor necrosis factor-alpha
TPOAb	anti-thyroid peroxidase antibodies
TSH	thyroid-stimulating hormone
TT3	total triiodothyronine
TT4	total thyroxine
VCAM-1	vascular cell adhesion molecule-1
VEGF	vascular endothelial growth factor
VUE	villitis of unknown etiology
