# Supplementary material for: Perinatal Endocrine–Cardiac Axis: A Narrative Review of Long-Term Cardiovascular Risks in Women with Gestational Diabetes, Hypertensive Disorders, and Thyroid Dysfunction
Source: Biomedicines. 2026 Jun 10;14(6):1322. doi: 10.3390/biomedicines14061322 (PMC13297138; doi:10.3390/biomedicines14061322)
Supplement: Supplementary file 1 [file biomedicines-14-01322-s001.zip › biomedicines-4357809-supplementary.pdf]

Supplementary Materials

Table S1 Core Screening Questions & Mandatory Postpartum Interventions (All Females ＜50 Years)

Three Key Pregnancy History Questions	Corresponding Mandatory Postpartum Actions (If Answer = Yes)
GDM?	Screen at 6–12 weeks postpartum (refer to main text Table 2)
HDP?	Annual regular monitoring of BP, glucose, lipids and TSH as clinical indicated
Thyroid dysfunction during pregnancy (hypothyroidism, hyperthyroidism, postpartum thyroiditis)?	Document all pregnancy-related endocrine complications in EHR as permanent CVD risk-enhancing factor

Table S2 Standard Postpartum & Long-Term Follow-Up Screening Schedule

Screening Time Node	GDM Related Screening Items	HDP Related Screening Items	Thyroid Disorder Related Screening Items
6–12 weeks postpartum	75g OGTT, blood lipids, blood pressure (BP)	Office & home BP monitoring; urinalysis if hypertension persists	TSH, FT4 detection
Annually thereafter	Blood glucose / HbA1c (recheck every 1–3 years), lipids, BP	BP, lipids, blood glucose (recheck every 1–3 years)	TSH detection (1–2 years after postpartum thyroiditis; follow permanent hypothyroidism management standard thereafter)

Table S3 First-Line Preventive Intervention Strategies by Medical History

Medical History Category	Universal Lifestyle Intervention (All High-Risk Women)	Optional Pharmacotherapy Intervention
GDM history	Maintain BMI 18.5–24.9 kg/m^2; adhere to Med/DASH diet; weekly exercise ≥150 minutes; exclusive breastfeeding ≥6 months	Metformin administration for prediabetes state (HbA1c 5.7–6.4%, or IFG/IGT confirmed)
HDP history	Same lifestyle requirements as GDM; daily sodium intake limited to ＜2300 mg	Low-dose aspirin for women with ≥2 additional CVD risk factors (HTN, diabetes, dyslipidaemia, CKD)
Thyroid dysfunction history	Same basic lifestyle requirements; strictly adhere to levothyroxine medication if prescribed	L-T4 replacement therapy to maintain TSH level at 0.5–2.5 mIU/L for permanent hypothyroidism

Table S4 Core Management Mnemonic & Early Referral Red Flags

4W Core Management Mnemonic	Early Cardio-Obstetrics Specialist Referral Red Flags
1. Women – Mandatory inquiry of all female patients' pregnancy history	1. Recurrent GDM or HDP (≥2 affected pregnancies)
2. Weeks – Standard screening implemented at 6–12 weeks postpartum	2. Pre-existing PCOS or metabolic syndrome
3. Way of life – Standardize diet, exercise, breastfeeding and weight management	3. Preterm preeclampsia (onset $<$34 weeks) or eclampsia
4. Who follows – Clear handoff to primary care team with permanent EHR risk alert	4. Persistent TSH abnormality lasting $>$6 months postpartum
—	5. Postpartum BP ≥140/90 mmHg detected at 6-week follow-up
—	6. Abnormal echocardiographic findings (LV hypertrophy, diastolic dysfunction, reduced GLS)
